# Olaparib modulates DNA repair efficiency, sensitizes cervical cancer cells to cisplatin and exhibits anti-metastatic property

**DOI:** 10.1038/s41598-017-13232-3

**Published:** 2017-10-09

**Authors:** Chandra Bhushan Prasad, Shyam Babu Prasad, Suresh Singh Yadav, Laxmi Kant Pandey, Sunita Singh, Satyajit Pradhan, Gopeshwar Narayan

**Affiliations:** 1 Department of Molecular and Human Genetics, Institute of Science, Banaras Hindu University, Varanasi, India; 2 Department of Obstetrics & Gynaecology, Institute of Medical Sciences, Banaras Hindu University, Varanasi, India; 3 Department of Zoology, Mahila Mahavidyalaya, Banaras Hindu University, Varanasi, India; 4 Department of Radiotherapy & Radiation Medicine, Institute of Medical Sciences; Banaras Hindu University, Varanasi, 221005 India

## Abstract

PARP1 trapping at DNA lesion by pharmacological inhibitors has been exploited in several cancers exhibiting defects in DNA repair mechanisms. PARP1 hyperactivation is involved in therapeutic resistance in multiple cancers. The role of PARP1 in cervical cancer (CC) resistance and implication of PARP inhibitor is yet to be elucidated. Our data demonstrates significantly higher expression of PARP1 in primary cervical tumors and CC cell lines SiHa and ME180. Upon cisplatin treatment CC cells display significant overexpression of PARP1 and its hyperactivation. PARP inhibitor olaparib shows significant anti-proliferative effect on CC cells and drive loss of clonogenic survival and enhanced cell death in combination with cisplatin. PARP inhibited cells show delay in resolution of γH2A.X foci and prolonged late S and G2-M phase arrest resulting in apoptosis. Further, PARP inhibition disrupts the localization of base excision repair (BER) effector XRCC1 and non-homologous end joining (NHEJ) proteins Ku80 and XRCC4. Due to disrupted relocation of repair factors, cisplatin induced stalled replication forks collapse and convert into double strand breaks (DSBs). Interestingly, PARP inhibition also shows anti-migratory and anti-invasive properties in CC cells, increases anchorage independent cell death and induces anoikis. Collectively, our data demonstrates therapeutic potential of PARP inhibitor in cervical cancer.

## Introduction

Among all the 17 members of PARP family, PARP1 is one of the most abundant proteins which is involved in regulation of transcriptional control, maintenance of genomic integrity, DNA repair and regulation of apoptotic and survival balance in cells^1,2^. PARP1 is abundantly localized in nucleus and 80% of its enzymatic activity includes PARylation of nuclear proteins, recruitment of DNA repair factors and stabilization of chromatin for transcriptional regulation^3^. In past 4 decades several potent PARP inhibitors have been discovered and clinically investigated as chemotherapeutic agent for the treatment of cancers with inherent defect in their DNA repair pathways^4^. Several PARP inhibitors including Olaparib (AZD2281), Niraparib (MK-4827), Veliparib and BMN673 have been identified as potential chemotherapeutic agents. These pharmacological inhibitors have shown antitumor activity alone or in combination with platinum based therapeutic agents in several cancers with DNA repair defects including ovarian^5-8^ and breast cancers^7–9^. Olaparib has been demonstrated to exert anticancer property in BRCA1 or BRCA2 deficient breast cancer^8,10^, loss of ERCC1 and synergistic interaction with cisplatin in non-small cell lung carcinoma^11–13^, ATM depletion in breast cancer^14^, MRE11 loss in endometrial cancer^15^ and defect in homologous recombination^16,17^. Olaparib also tends to increase oncolytic activity of dl922-947 in a model of anaplastic thyroid carcinoma^18^. Contribution of PARP1 in regulation of metastatic events has also been thoroughly investigated in DNA repair independent manner in lung cancer via S100A4 and in melanoma via regulation of vimentin expression^19–21^. Recent phase I trial of PARP inhibitor in combination with cisplatin and paclitaxel shows well tolerance and promising results in both persistent and recurrent cervical cancer^22^.

Platinum based chemotherapeutic agents are the mainstream treatment line for most of the solid tumors. Platinum resistance is one of the biggest hurdles in successful treatment of tumors. Several lines of evidence reveal that PARP is responsible for platinum resistance in cancer^23^. PARP1 is able to detect DNA strand nicks and binds to it. After binding with DNA strand PARP1 undergoes PARylation and detach from the DNA, olaparib inhibits the auto-modification of PARP1 and traps it on the DNA strand causing inhibition of its enzymatic activity^24^. PARP1 trapping at damaged DNA strand inhibits the recruitment of DNA repair enzymes at the site leading to creation of persistent double strand breaks (DSBs) in DNA which is more lethal compared with depletion of PARP protein^25^. PARP plays potential role in regulation of homologous recombination (HR) and NHEJ^26,27^, competes with Ku70/80 for DNA binding in NHEJ pathway^28^ and regulation of XRCC1 recruitment during oxidative stress and other genomic insults^29,30^.

Our results suggest that PARP inhibition by olaparib and its combination with cisplatin has profound anticancer effect and anti-metastatic effect and may be used as therapeutic strategy in treatment of advanced cervical carcinoma.

## Results

### CC cells exhibit higher amount of PARP1

**Figure 1:**
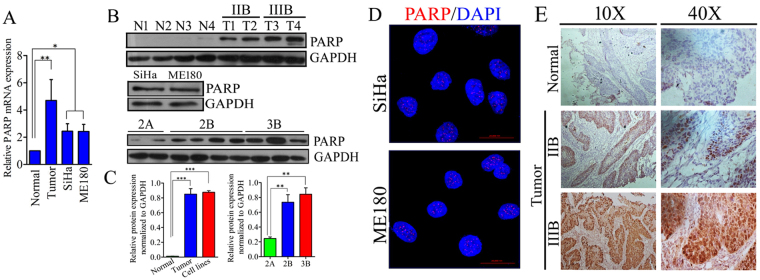
Expression of PARP1 in primary tissue samples and CC cell lines; (**A**) Bar graph displaying quantitative mRNA expression of PARP1 in normal samples (n = 15) and tumor samples (35) along with two cervical cancer cell lines SiHa and ME180. (normal *vs*. tumor **p = 0.0035, normal *vs*. cell lines *p = 0.0359, tumor *vs*. cell lines *p* = 0.3993. (**B**) Western blots displaying expression of PARP1 protein in normal and tumor samples as well as in CC cell lines.The full size blots corresponding to the cropped blot images are given in Supplementary Figure S12. (**C**) Bar graph showing relative quantification of protein expression normalized to GAPDH. (**D**) Representative immunofluorescence image showing nuclear localization of PARP1 as foci in SiHa and ME180 cell lines. (**E**) Representative image showing nuclear PARP1 expression in tumor samples from CC patient. Normal samples do not show any nuclear staining. However, IIIB samples have greater staining intensity than IIB.

We investigated the expression pattern of PARP1 in CC cell lines and primary tissue samples using quantitative real-time PCR and western blot analysis from total RNA and protein respectively. Our results show significant upregulation of PARP1in tumor samples and in CC cell lines compared with normal cervical tissue both at transcript and protein levels. PARP1 protein is also highly expressed in IIB and IIIB stages of the tumor in comparison to IIA stage. Advancement of the disease from IIB to IIIB displayed trend of upregulation in PARP1 expression, however the difference is not significant (Fig. 1A–C). CC cell lines SiHa and ME180 showed nuclear accumulation of PARP1 foci (Fig. 1D). Next, we investigated PARP1 expression pattern in pre-chemotherapeutic, formalin fixed samples from CC patients. PARP1 found to be express in primary tumors and have greater nuclear intensity in IIIB samples than IIB samples, however normal sample show a little expression in nucleus (Fig. 1E).

### Cisplatin mediated replication stress trigger PARP1 over-expression & hyperactivation in CC cell lines

**Figure 2:**
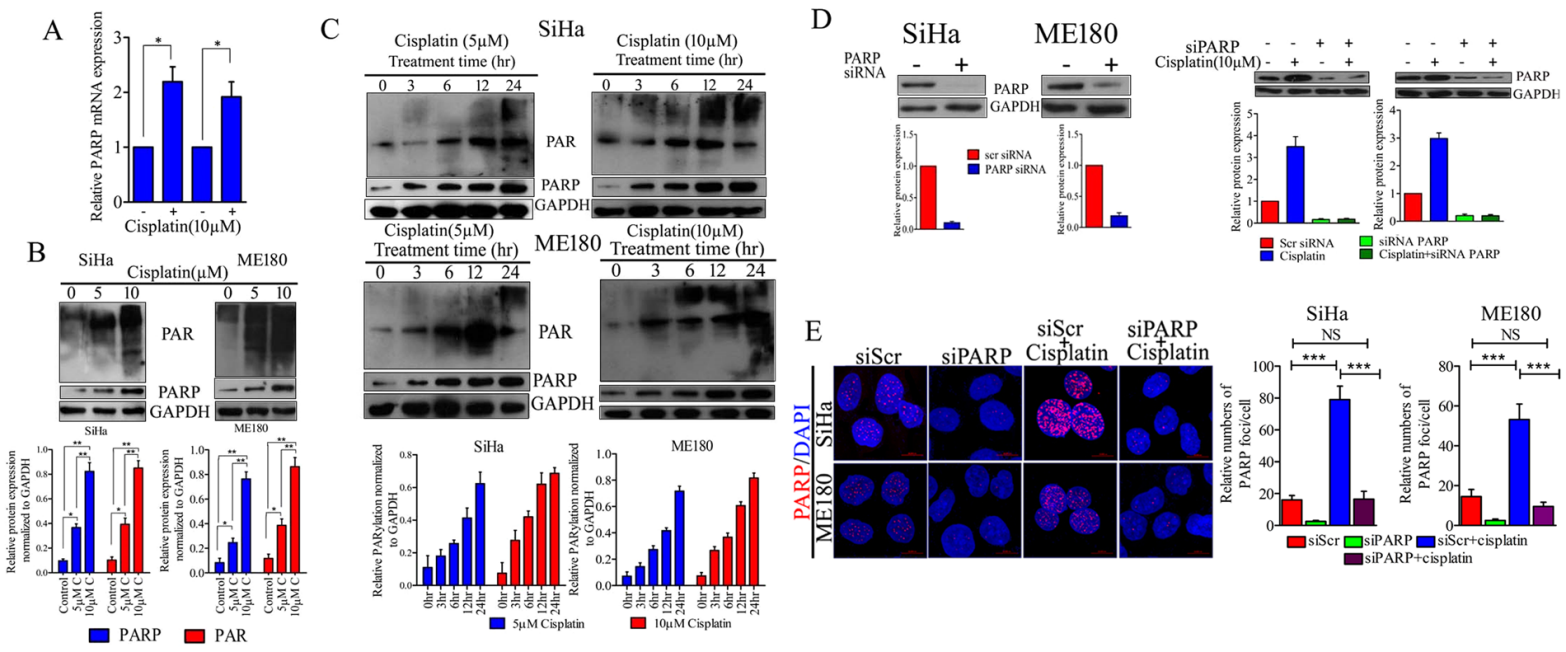
Cisplatin induces PARP1 over expression and hyperactivation in CC cell lines. (**A**) Graph showing relative PARP1 mRNA expression in SiHa and ME180 CC cell lines after 24 hr cisplatin (10μM) treatment. (SiHa control vs. cisplatin *p = 0.0205, ME180 control vs. cisplatin *p = 0.0351). (**B**) Representative western blot showing expression of PARP1 and PAR polymers in SiHa and ME180 cell lines treated with 5 µM and 10 µM cisplatin for 48 hrs. Bar graph displaying normalized band density of PARP1 and PAR normalized to GAPDH. Error bar display the S.D. from the mean of 3 independent experiments. *p = 0.01,**p = 0.007. The full size blots corresponding to the cropped blot images are given in Supplementary Figure S12. (**C**) Western blot showing PARP1 and PAR expression in SiHa and ME180 cell lines treated with 5µM and 10µM for indicated time duration. Bar graph displaying normalized band density of PARP1 and PAR normalized to GAPDH band intensity of respective blot image. The full size blots corresponding to the cropped blot images are given in Supplementary Figure S12. (**D**) Transfection with specific PARP1 siRNA shows depletion in basal PARP1 protein level as well as cisplatin induced PARP1 overexpression. Bar graph displaying relative protein expression normalized to GAPDH. The full size blots corresponding to the cropped blot images are given in Supplementary Figure S12. (**E**) Representative immunofluorescence image showing nuclear localization of PARP1 in SiHa and ME180 cell lines. 24 hrs cisplatin treated cell shows enhanced number of PARP1 foci in both cell lines. Bar graph displaying quantitative distribution of PARP1 foci in respective treatment groups.

We and others have previously shown that platinum-DNA adduct formation by cisplatin treatment causes significant replication stress in CC cells as well as in other cancer cell types^31–33^. Replication stress caused by cisplatin triggered overexpression of PARP1 in both the SiHa and ME180 cell lines at transcript and protein levels (Fig. 2A and B). Cisplatin treatment also triggers the hyperactivation of PARP1 which was measured by detection of PAR polymers. In order to assess the kinetics of PARP1 overexpression and PAR formation, CC cells were incubated with two different concentrations (5 µM and 10 µM) of cisplatin for 48 hrs or for indicated time points (3, 6, 12, 24 hrs) and expression of PARP1 and PAR was determined by western blot analysis. PARP1 is overexpressed and activated in response to cisplatin dose- dependently. Time dependent formation of PAR was also evaluated simultaneously in CC cell lines. Cisplatin causes PARP1 hyperactivation in both SiHa and ME180 cell lines. PAR polymers formation was started as early as after 3 hr treatment of cisplatin. The level of PAR polymers increases with cisplatin concentration and treatment duration (Fig. 2C). Similarly, depletion of PARP1 with specific siRNA was also able to reduce basal PARP1 protein level as well as cisplatin induced protein elevation of PARP1 in both the cell lines (Fig. 2D). SiHa and ME180 cell lines also show higher nuclear accumulation of PARP1 foci after cisplatin treatment (Fig. 2E). Elevation in PAR polymer level clearly indicates the hyperactivation of PARP1 in CC cells in response to replication stress caused by platinum-DNA adduct.

### Effect of Olaparib on cisplatin mediated hyperactivation of PARP1

**Figure 3:**
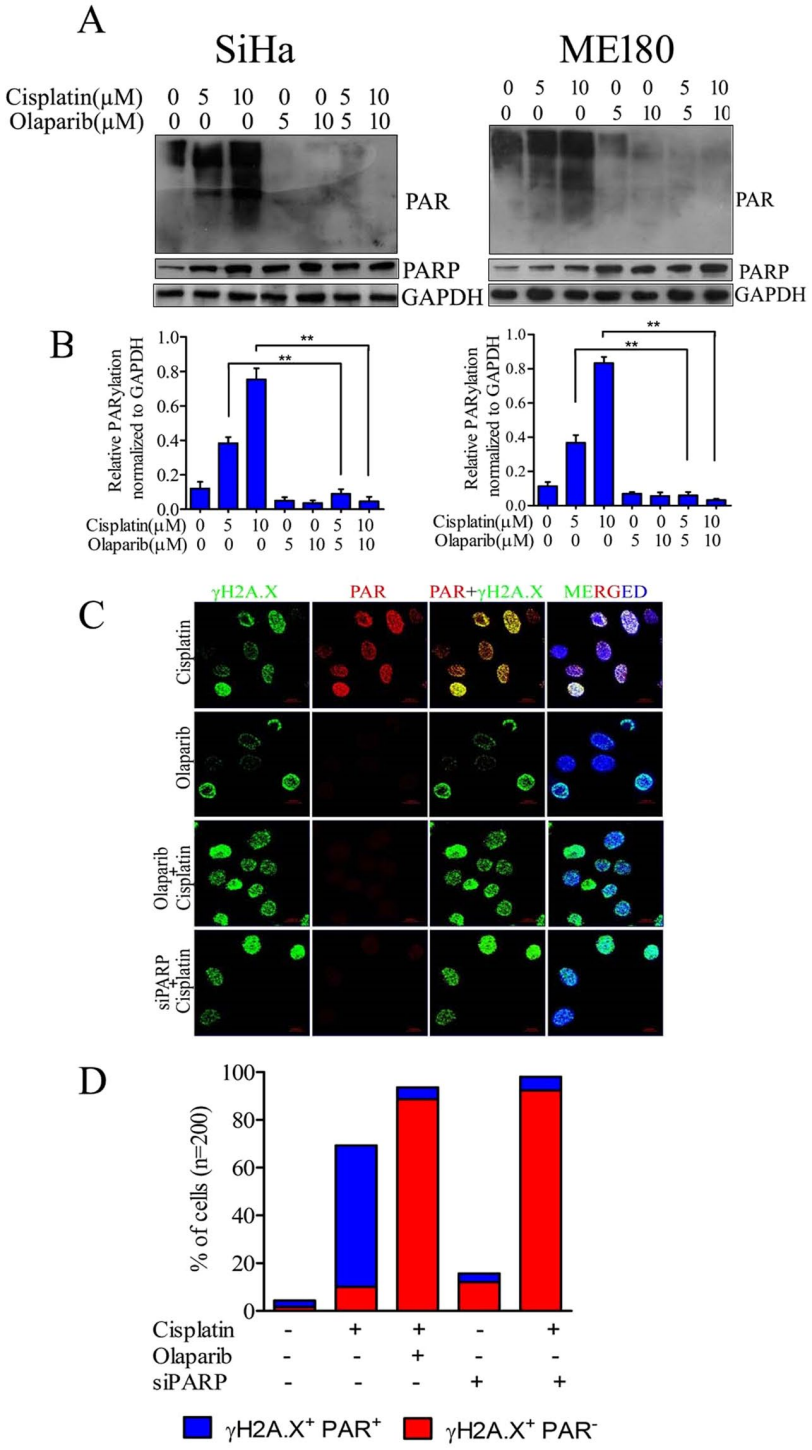
Effect of olaparib on cisplatin mediated PARP1 hyperactivation. (**A**) SiHa and ME180 cells were treated with cisplatin and/or olaparib for 48 hrs and expression of PARP1 and PAR was determined by western blot. Olaparib exerts strong anti-PARP activity in cisplatin treated CC cells.The full size blots corresponding to the cropped blot images are given in Supplementary Figure S12. (**B**) Bar graph displaying relative PARylation normalized to GAPDH in both cell lines. Error bars display the S.D. from the mean of 3 independent experiments. **p = 0.0095. (**C**) Representative immunofluorescence image showing nuclear co-localization of PAR polymers with γH2A.X foci. 24 hr cisplatin treated cells show higher PAR polymers signals compare to untreated and signal was completely suppressed after olaparib treatment or PARP1 siRNA transfection. (**D**) Bar graph displaying quantification of γH2A.X^+^ PAR^−^ or γH2A.X^+^ PAR^+^ cell distribution in cisplatin and/or olaparib treated along with PARP1 silenced cells treated with cisplatin.

Earlier reports indicate the involvement of PARP1 hyperactivation in cisplatin resistance in other tumor types.To determine the effect of olaparib on PARP1 activity, CC cells were treated with cisplatin along with two different concentrations (5 µM and 10 µM) of olaparib. Results demonstrate that olaparib treatment was sufficient to inhibit cisplatin induced formation of PAR polymers (Fig. 3A and B). PARP1 hyperactivation was also confirmed with immuno-staining of PAR polymers in cells which show significant nuclear accumulation in cisplatin treated cells than untreated cells. PAR polymer formation was completely suppressed by olaparib in cisplatin treated cells in both the cell lines. Similarly, PARP1 siRNA transfected cells show inhibition of PAR formation in response to cisplatin (Fig. 3C). PARP1 inhibited or silenced cells in combination with cisplatin exhibit profound DNA damage and produce more numbers of γH2A.X^+^ PAR^−^ cells compare to γH2A.X^+^ PAR^+^ cells produced by cisplatin alone. CC cells treated with combination of cisplatin and olaparib displayed higher number of γH2A.X foci than single agent (Fig. 3D)

### Olaparib increases cisplatin induced lethality in CC cells

**Figure 4:**
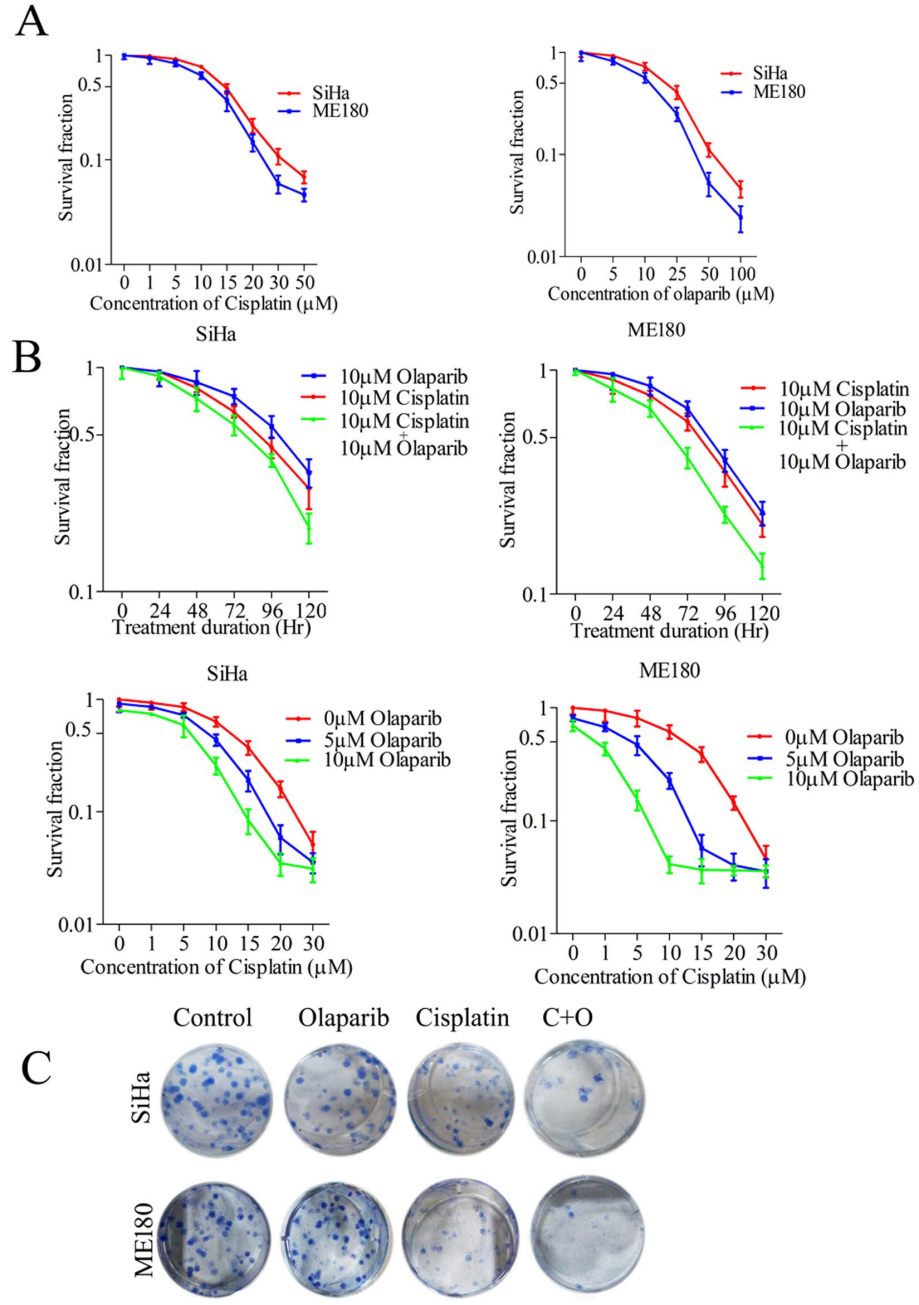
Olaparib increases cisplatin induced lethality in CC cells. (**A**) Cell proliferation assay was carried out to determine the anti-proliferative potential of cisplatin and olaparib in indicated concentration for 72 hrs drug incubation and proliferation was assessed by MTT assay. Data indicate that both the drugs were able to inhibit cellular proliferation of CC cell lines SiHa and ME180. (**B**) Both the cell lines were treated with cisplatin and/or olaparib for indicated duration or indicated drug concentration of each drugs, cell proliferation was determined by MTT assay. Data indicates that olaparib significantly augment the cisplatin induced cell cytotoxicity in CC cell lines. (**C**) Olaparib treatment significantly enhanced cisplatin induced loss of clonogenic survival of CC cells.

An earlier report shows that PARP inhibitors trap PARP1 molecules at the site of DNA damage and inhibit its detachment from DNA strand^24^. SiHa and ME180 cells were treated with indicated concentrations of cisplatin or olaparib alone for 72 hrs and cell proliferation was estimated using MTT assay. Cell proliferation assay showed that cisplatin and olaparib inhibit cell proliferation significantly at higher drug doses (Fig. 4A). Since CC cells produce more PARP1 in response to cisplatin, we introduced PARP inhibitor along with cisplatin to trap more PARP1 molecules at DNA damage sites. Cells treated with cisplatin (10 µM) and olaparib (10 µM) show significant inhibition of cell growth than cells treated with a single drug. Further, cisplatin was also combined with two different doses (5 µM and 10 µM) of olaparib and cell proliferation was investigated after 72 hrs of treatment. Combination of drugs was able to inhibit cell proliferation more potentially than a single drug (Fig. 4B). Similarly, prolonged clonogenic survival also displayed that cisplatin-induced loss of clonogenic survival was increased by simultaneous treatment of olaparib in both the CC cell lines (Fig. 4C). Combination of Cisplatin and Olaparib did not show significant increased inhibition of cell survival in HEK239 cells (Supplementary Figure S1). Results suggest that olaparib trap higher amounts of PARP1 induced by cisplatin and simultaneously exert enhanced lethality in CC cells.

### PARP inhibition collapses cisplatin induced stalled replication fork and produce more DSBs

**Figure 5:**
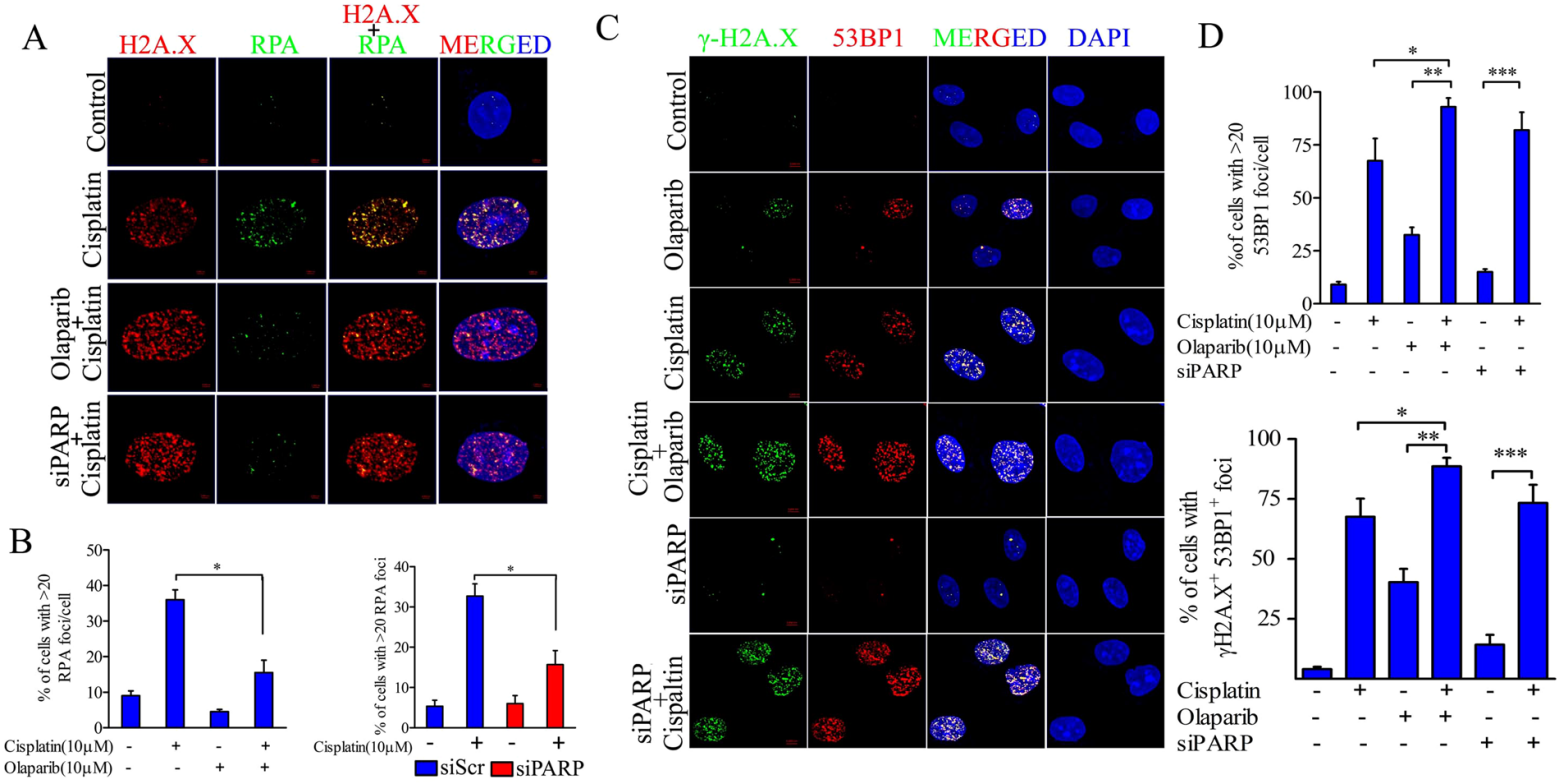
PARP1 inhibition stalls replication fork and induces DSBs. (**A**) Representative immunofluorescence of RPA and γH2A.X foci in cisplatin and/or olaparib treated cell CC cell lines along with PARP1 silenced cell treated with cisplatin. (**B**) Quantification of percentage of cells with >20 RPA foci/cell in indicated treatment groups (cisplatin *vs*. cisplatin + olaparib and PARP1 siRNA *vs*. PARP1 siRNA + cisplatin *p < 0.05). (**C**) Representative immunofluorescence of 53BP1 foci and γH2A.X foci in indicated treatment or siRNA transfection in CC cells. Cisplatin and olaparib both induce 53BP1 foci in CC cell line but combination of both drug or cisplatin treatment in PARP1 depleted cells produces higher number of 53BP1 foci and display more number of γH2A.X foci which co-localizes with each other. (**D**) Quantification plot showing percentage of cells showing >20 53BP1 foci in mentioned treatment groups and γH2A.X^+^53BP1^+^cells in respective mentioned groups.

Platinum salt based therapeutic agents are well known for their replication stress via formation of platinum-DNA adducts in dividing cells. Therefore, we investigated the potential of PARP1 inhibition and progression of replication fork caused by therapeutic agent cisplatin. Replication protein A (RPA) binds to single stranded DNA (ssDNA) and actively facilitates the replication. RPA foci serve as marker for active replication fork as well as ssDNA. Cells were treated with cisplatin and/or olaparib for 24 hrs and number of RPA foci was observed in both the groups. Cells treated with cisplatin show significantly higher RPA foci which were relocated at replication fork stalled sites. Cisplatin induced RPA foci were significantly lower in PARP1 inhibited cells but cells exhibit higher number of γH2A.X foci. Similarly, PARP1 depleted cells also show reduced number of RPA foci and higher number of γH2A.X foci after cisplatin treatment (Fig. 5A and B). This suggests the active role of PARP1 in formation of ssDNA and active replication fork. PARP1 inhibited and silenced cells show rapid induction of γH2A.X foci and complete relocation of DSB repair effector 53BP1 foci at damage sites. Cells treated with either cisplatin or olaparib displayed less number of γH2A.X and 53BP1 foci than the combined treatment. DNA damage induced by cisplatin in PARP1 inhibited or silenced background produces higher number of 53BP1 foci and display higher number of cells positive for γH2A.X^+^53BP1^+^ (Fig. 5C and D).

### PARP1 inhibition/silencing enhances cisplatin sensitivity in CC cells and rapid induction of DNA damage

**Figure 6:**
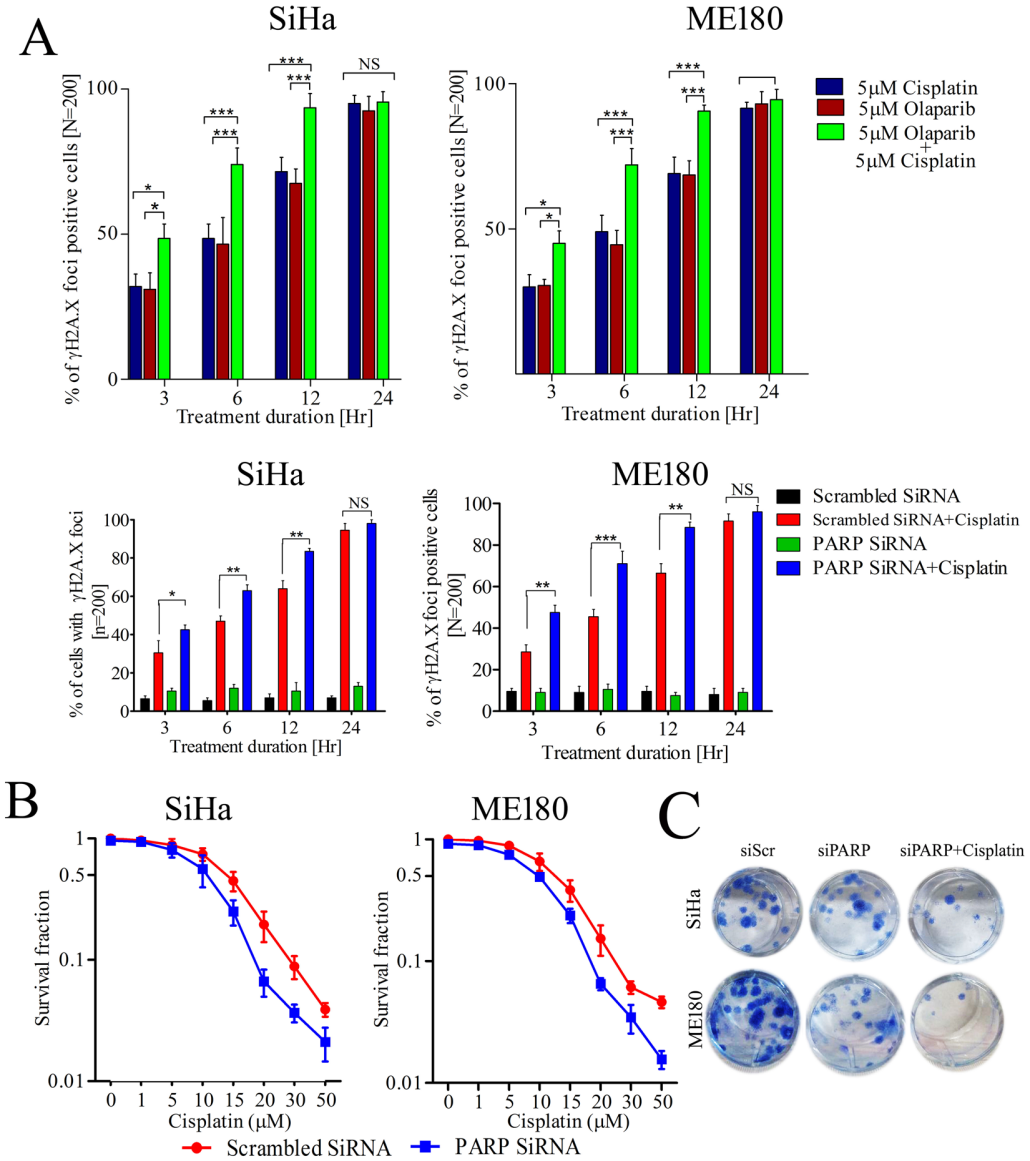
PARP1 inhibition sensitizes CC cells and leads to rapid induction of DNA damage and cell death. (**A**) Bar graph showing the percentage of γH2A.X foci positive cells (cell with >20 positive foci/cell as compared to isotype) in indicated groups at particular treatment duration. At least 200 cells were counted in blind fashion in independent duplicate experiment. Error bar show SD from the mean of two independent counts. *p = 0.05, **p < 0.01, ***p < 0.001 (upper panel); Bar graph showing the percentage of γH2A.X foci positive cells in PARP1 silenced and control counterpart cells treated with 5 µM cisplatin for indicated time points. At least 200 cells were counted in blind fashion in independent duplicate experiment. Error bar display SD from the mean of two independent counts. *p = 0.05, **p < 0.01, ***p < 0.001 (lower panel). (**B**) Cell proliferation assay displaying effect of cisplatin on PARP1 silenced CC cells. (**C**) PARP1 silenced cells showing increased loss of clonogenic survival after cisplatin treatment in CC cells.

To assess the DNA damage capability of cisplatin in PARP1 inhibited cells, CC cell lines were treated alone or in combination with both the drugs. Combined treatment was able to produce higher number of cells positive for γH2A.X signals than either of the single drug. PARP inhibitor olaparib significantly exacerbated cisplatin induced DNA damage in CC cells. Cells with positive γH2A.X foci were not significant after 24hr of treatment since there were more frequent DNA damage sites in the cells (Fig. 6A). Similarly, PARP1 silenced SiHa and ME180 cells show rapid induction of γH2A.X foci (Fig. 6B). PARP1 silenced CC cells and scrambled counterparts were exposed to different concentration of cisplatin for 72 hrs. Cell proliferation assay displayed significant reduction in cell survival in PARP1 depleted cells.

### PARP1 inhibited cells display significant delay in DNA repair and cell cycle progression

**Figure 7:**
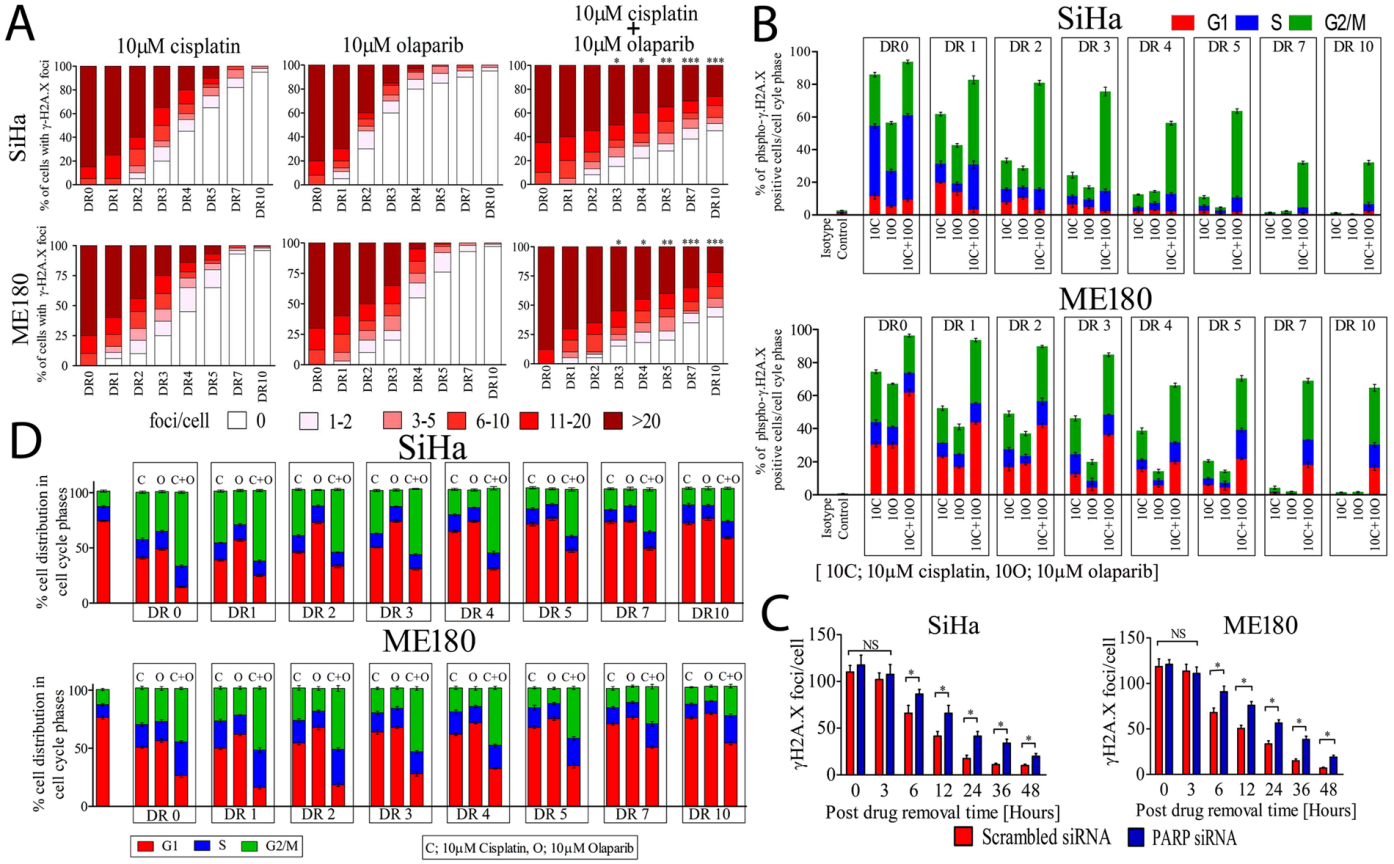
PARP1 inhibition delayed DNA repair and leads to prolonged cell cycle arrest at G2/M. (**A**) Both the cell lines SiHa and ME180 were treated with 10 µM cisplatin and/or olaparib for 24 hrs. After 24 hrs drug was removed (DR0) and fresh drug free media was added. Cells were cultured further and γH2A.X foci were observed very 24 hr till 10 days post drug removal (DR0 to DR10). Cells with >20 γH2A.X foci to 0 foci were counted in blind fashion and at least 200 cells per group in several different 60× objective fields were acquired. Quantitative plot displaying distribution of γH2A.X foci in three treatment groups in both the cell lines. [Time point DR3, frequency of cells with more than 20 foci per cell in SiHa; cisplatin vs. cisplatin + olaparib *p = 0.0467, at DR4; cisplatin vs. cisplatin + olaparib *p = 0.0268, at DR5; cisplatin vs. cisplatin + olaparib **p = 0.0060 and frequency of cells with more than 20 foci per cell in ME180 at DR3; cisplatin *vs*. cisplatin + olaparib *p = 0.0390, at DR4; cisplatin *vs*. cisplatin + olaparib *p = 0.041, at DR5; cisplatin *vs*. cisplatin + olaparib **p = 0.0024]. **(B**) Both the cell lines SiHa and ME180 were treated as indicated and cell were stained with FITC tagged anti γH2A.X & co-stained with PI and analysis was done using FACS for DNA damage in each cell cycle phases. Graph showing the percentage of cells with γH2A.X foci in SiHa and ME180 cell lines. PARP1 inhibition in cisplatin treated cells shows significant accumulation of DNA damage in late S and G2/M phase compared to G1. (**C**) Graph showing frequency of γH2A.X foci resolution in PARP1 silenced and control counterpart after cisplatin treatment. PARP1 silenced cells displayed significant delay in γH2A.X foci resolution after drug removal. (**D**) Similarly, PARP inhibition along with cisplatin treatment for 48 hrs shows significant & prolonged G2/M and late S phase arrest in both the cell lines after drug removal as in previous experiments.

DNA repair kinetics was carried out for 10 days after single or combined treatment with cisplatin (10 µM) and/or olaparib (10 µM) for 24 hrs. Both cell lines were exposed to drugs for 24 hrs and drugs were removed (DR); at time point 0 (DR0) cells were fixed and cells were observed for γH2A.X foci every 24 hrs till 10 days (DR0 to DR10). The frequency of cells with γH2A.X foci was counted in all three groups in both the cell lines. After 24 hrs of drug treatment, frequency of γH2A.X foci increased in all the three groups. 70–80% of the cells had more than 20 foci regardless of drug treatment groups. After drug removal there was no significant difference in resolution of γH2A.X foci till 48 hrs. In contrast, PARP1 inhibited cells treated with cisplatin show significant delay in γH2A.X foci resolution than either olaparib or cisplatin treatment [Time point DR3, frequency of cells with more than 20 foci per cell in SiHa; cisplatin vs. cisplatin + olaparib *p* = 0.0467, at DR4; cisplatin vs. cisplatin + olaparib *p* = 0.0268, at DR5; cisplatin vs. cisplatin + olaparib *p* = 0.0060 and frequency of cells with more than 20 foci per cell in ME180 at DR3; cisplatin *vs*. cisplatin + olaparib *p* = 0.0390,at DR4; cisplatin *vs*. cisplatin + olaparib *p* = 0.041, at DR5; cisplatin *vs*. cisplatin + olaparib *p* = 0.0024]. PARP1 inhibited cells fail to resolve the global DNA damage induced by cisplatin in both the cell lines even after 10 days of drug removal (Fig. 7A and Supplementary Figure S2). Total cells with γH2A.X foci were also analyzed in same experimental condition in both the cell lines. Cells were co-stained with PI and FITC tagged γH2A.X and 10,000 cells were acquired by flow cytometer. PARP1 inhibited cells showed significant amount of DNA damage in every phase of cell cycle in time course analysis of DNA repair. Combined treatment shows increased DNA lesions in late S and G2/M phase than G1 phase (Fig. 7B and Supplementary Figure S3). We also assessed the DNA repair capacity of PARP silenced cells in both the cell lines. We exposed both PARP1 silenced and control cells to 5 μM cisplatin for shorter duration (3 hr) and then drug was replaced with drug free medium and cells were analyzed for γH2A.X foci resolution. PARP1 silenced cells failed to repair DNA damage and show significant delay in resolution of γH2A.X foci than the cells transfected with scrambled siRNA (Fig. 7C and Supplementary Figure S4).

We also investigated the cell cycle response of cisplatin and/or olaparib treated SiHa and ME180 cells. As described in previous experiments, cells were treated with either single drug or in combination of drugs and were analyzed for cell cycle in flow cytometer. Although, cisplatin and olaparib both were able to arrest the cell cycle in G2/M phase as single agent, PARP1 inhibition in DNA damage background exert profound and prolonged cell cycle arrest in late S and G2/M phase (Fig. 7D and Supplementary Figure S5).

### PARP1 inhibition interferes with recruitment of BER effector XRCC1 and NHEJ mediators XRCC4 and KU80 at DNA damage sites

**Figure 8:**
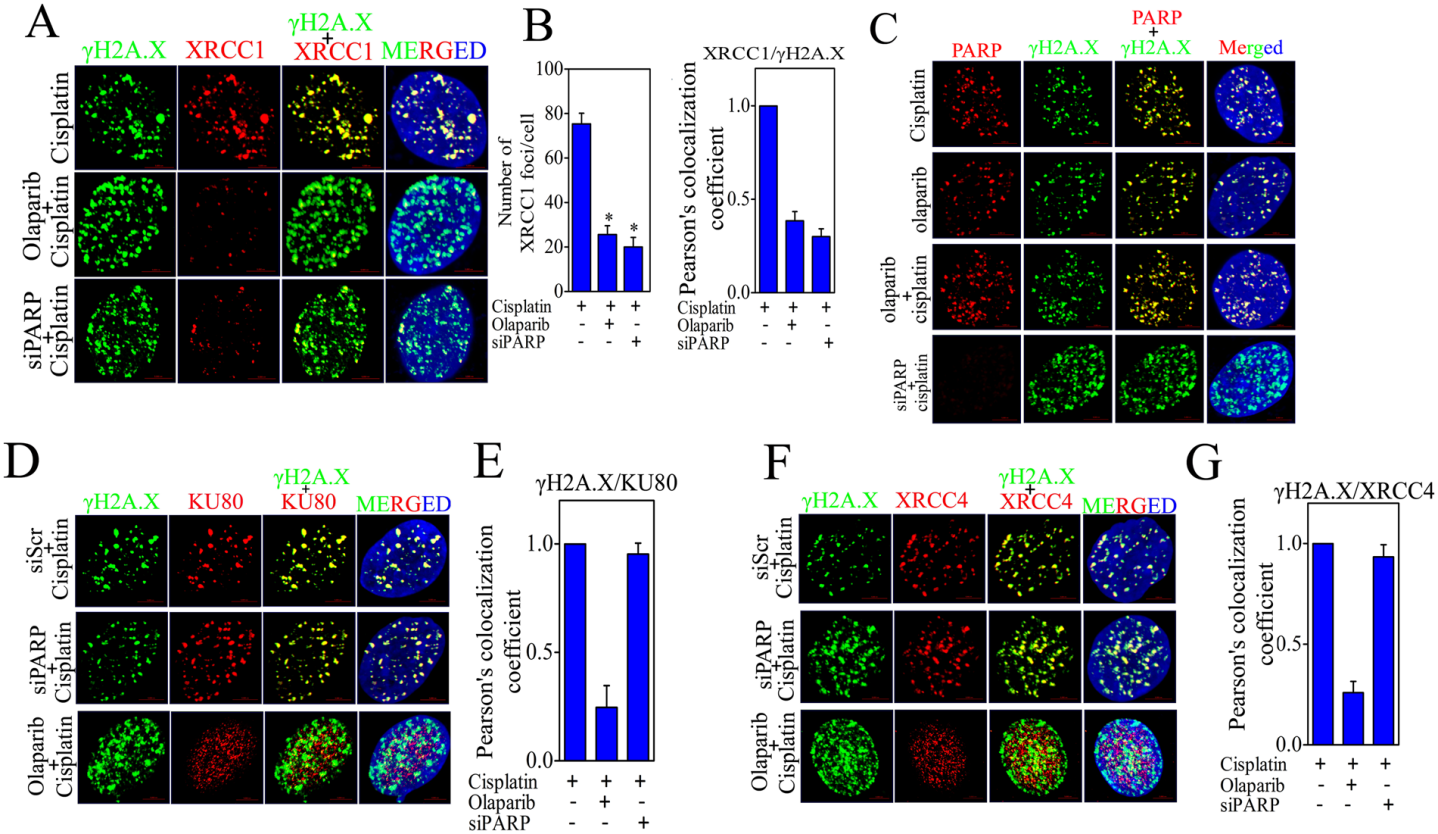
PARP1 inhibition interferes with recruitment of BER effector XRCC1 and NHEJ mediators XRCC4 & KU70/80 at DNA damage site. (**A**) Representative image of co-immunofluorescence of XRCC1 and γH2A.X in CC cell line treated with cisplatin and/or olaparib and siRNA as indicated. (**B**) Bar graph displaying number of XRCC1 foci and relative co-localization of XRCC1 foci and γH2A.X foci in indicated treatment group. (**C**) Representative image of co-immunofluorescence of PARP1 and γH2A.X in CC cell line treated with cisplatin and/or olaparib and siRNA as indicated. (**D**) Representative image of co-immunofluorescence of KU80 and γH2A.X in CC cell line treated with cisplatin and/or olaparib and siRNA as indicated. (**E**) Bar graph displaying relative co-localization of KU80 foci and γH2A.X foci in indicated treatment group. (**F**) Representative image of co-immunofluorescence of XRCC4 and γH2A.X in CC cell line treated with cisplatin and/or olaparib and siRNA as indicated. (**G**) Bar graph displaying relative co-localization of XRCC4 foci and γH2A.X foci in indicated treatment group.

We further investigated the result of PARP1 trapping at chromatin and its effect on BER effector XRCC1 and NHEJ mediators Ku80 and XRCC4 recruitment at DNA damage site. Co-immunofluoresence of γH2A.X was carried out with PARP1, XRCC1, XRCC4 and Ku80 in cisplatin, cisplatin + olaparib and PARP siRNA transfected CC cells treated with cisplatin. BER effector XRCC1 requires active PARP1 to be recruited at DNA damage sites and PARP inhibitors induces apoptosis in XRCC1 dependent manner^29,30^. We investigated the recruitment of XRCC1 in PARP1 inhibited CC cells. PARP inhibitor significantly reduces the XRCC1 foci formation and disrupts the relocation of XRCC1 at γH2A.X foci in CC cells. Similarly, PARP1 silenced cells also displayed disruption of XRCC1 relocation at damage site and significantly show lower number of XRCC1 foci (Fig. 8A and B). This suggests that presence of PARP1 protein as well as its activation is critical for XRCC1 recruitment at DNA damage sites. However, specific depletion of PARP1 with siRNA did not alter the relocation of XRCC4 and KU80 at damage sites but the trapping of PARP with olaparib significantly reduces the relocation of NHEJ mediators at DSBs sites (Fig. 8C–F). Since both PARP1 and KU80 proteins have high competition for DNA end binding, PARP1 trapping at DNA damage sites block the relocation and binding of NHEJ effectors resulting in sustained DSBs. Since PARP1 inhibited cells display enhanced DSBs, we further checked whether these DSBs act as substrate for HR mediated repair mechanism or not. Co-localization results show that the HR regulator RAD51 completely localizes with DSBs created by PAPR inhibition (Supplementary Figure S6).

### PARP1 inhibition/silencing enhance cisplatin induced apoptosis in cervical cancer cells

**Figure 9:**
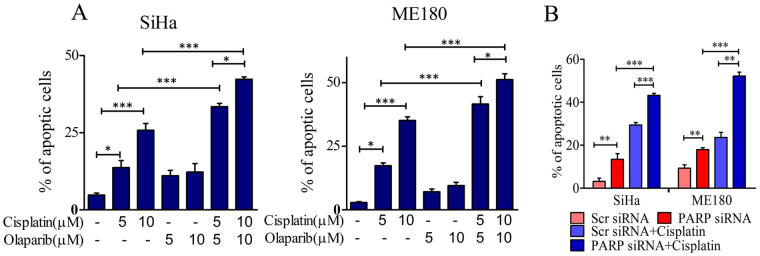
PARP1 inhibition or silencing enhances cisplatin induced apoptosis in cervical cancer cells. (**A**) Bar graph showing quantification of apoptotic cells in cisplatin and/or olaparib treated cells in both the cell lines. (**B**) Bar graph showing quantification of apoptotic cells in PARP1 silenced CC cells treated with cisplatin.

We explored the apoptotic efficacy of PARP1 inhibition in CC cells. To test the role of PARP1 silencing or inhibition on apoptosis, cells were exposed to cisplatin and/or olaparib for 24 hrs and apoptosis assay was carried out by Annexin V/PI co-staining. Olaparib and cisplatin both were able to induce apoptosis in CC cells in dose dependent manner but the combination of both the drugs exerts significantly higher apoptosis (Fig. 9A and Supplementary Figure S7). Similarly, PARP1 silencing also made CC cells more sensitive towards cisplatin and exert increased apoptosis (Fig. 9B and Supplementary Figure S8).

### PARP inhibitor suppresses metastatic events in CC

**Figure 10:**
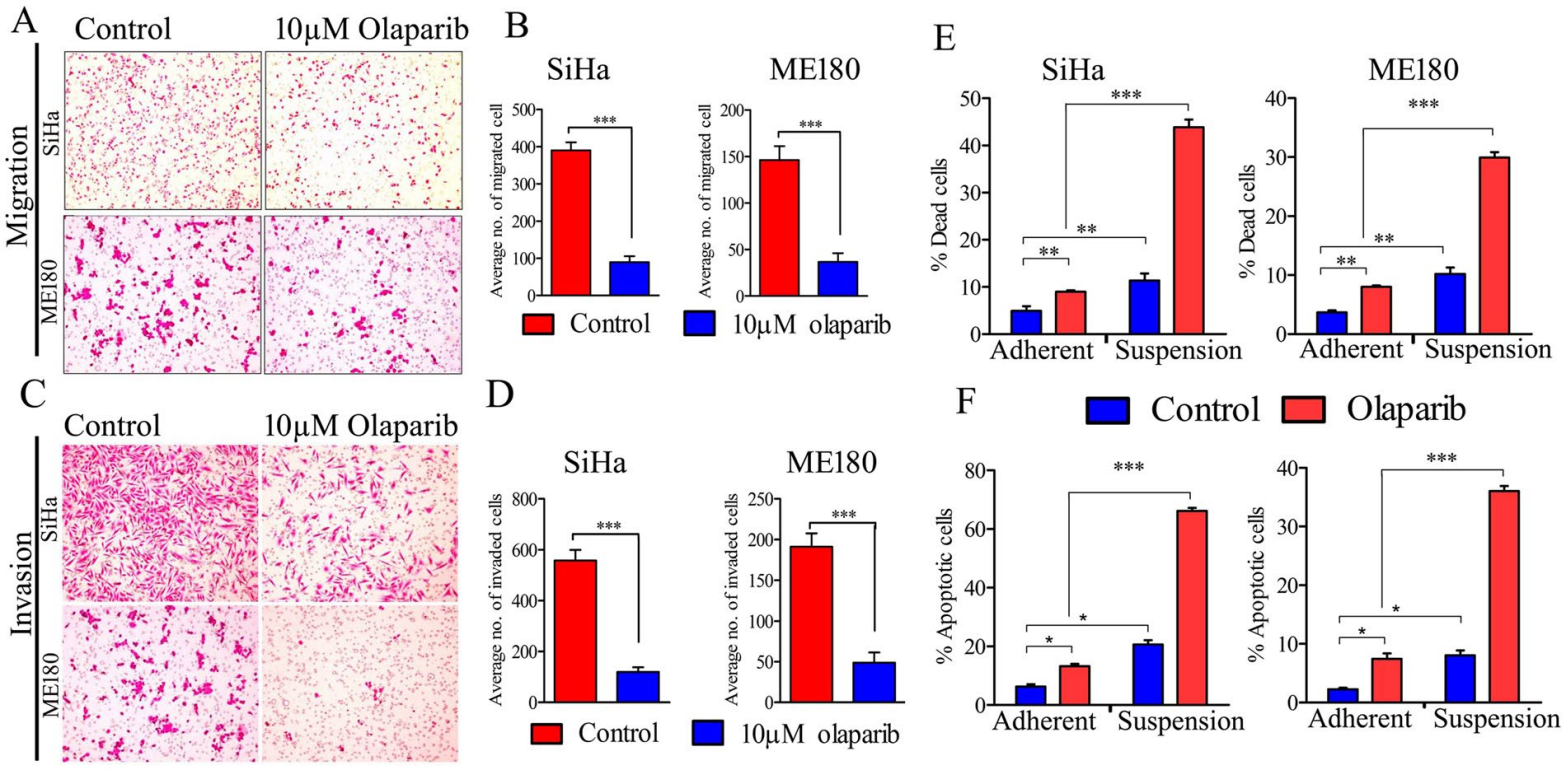
PARP inhibitor suppresses metastatic events in CC. (**A**) Overnight serum starved SiHa and ME180 cell lines were seeded in transwell insert (8 µm pore size) with or without olaparib for 16 hrs. Representative images show migrated cells stained with crystal violet. (**B**) Bar graph showing quantitative cell migration in untreated and olaparib treated CC cell lines. (**C**) Overnight serum starved SiHa and ME180 cell lines were seeded in transwell insert (8 µm pore size) coated with matrigel matrix and 10 µM olaparib was added with the cells for 48 hrs. Representative image shows invaded cells stained with crystal violet. (**D**) Bar graph showing quantitative cell invasion through matrigel matrix in untreated and olaparib treated CC cell lines. (**E**) SiHa and ME180 cell were cultured in adherent and suspension condition. Olaparib treatment was done for 24 hrs and cells were stained with PI. Bar graph showing the quantitative distribution of dead cells. (**F**) Olaparib treated adherent and suspension CC cell lines were co-stained with Annexin V and PI. Bar graph displaying quantitative distribution of apoptotic cells.

Several lines of evidence suggest that PARP regulates metastatic events in other cancers via pathway(s) independent of DNA repairs^19–21^. However, the role of PARP1 in CC metastasis is not documented yet. We investigated the effect of olaparib on metastatic events such as cell migration, invasion and anchorage independent cell survival of CC cells. Sub-lethal dose (IC50; 18.4 μM ± 1.5 μM for SiHa and 15.8 µM ± 1.2 µM for ME180) of olaparib (10 µM) displayed significant decrease in migration and invasion capacity of CC cell lines (Fig. 10A,B,C and D). To eradicate drug toxicity induced inhibition of cell migration and invasion, cells were stained with CFSE [(5(6)-Carboxyfluorescein N-hydroxysuccinimidyl ester)] stain and migration and invasion assay was performed. Healthy and live cells uptake CFSE and intracellular esterases cleave the acetate group resulting in green florescence. Olaparib treated cells show decrease in cell migration and invasion and was concordant with previous results and olaparib treated SiHa cells show decreased level of vimentin protein after olaparib treatment as well. (Supplementary Figure S9). This suggests that sub-lethal dose of olaparib was able to inhibit both migration and invasion of CC cells without altering cellular health or drug toxicity. Thereafter, effect of PARP inhibitor was investigated for targeting anoikis resistance. CC cell lines were cultured in both anchorage (adherent monolayer) and anchorage independent (suspension) conditions. Olaparib alone induces significant cell death in anchorage independent CC cells than adherent cells and promote significant apoptosis in suspension cells than in adherent CC cells (Fig. 10E and F and Supplementary Figures S10 and S11).

## Discussion

One of the major challenges in the era of cancer treatment is the identification of the pathways or genes involved in the development of resistance to conventional therapies i.e., radio-resistance or chemo-resistance. Although, some reports have identified some of the pathway(s) and molecular mechanisms responsible for cisplatin resistance in several cancers including CC, a comprehensive mechanism(s) is largely unclear^34–41^. Several markers have already been identified that collaborate with PARP inhibitor sensitivity in cancer cells^42–44^. Here, we have provided insight of molecular mechanism by which PARP inhibitor potentially collaborates with cisplatin in CC cells and exert anticancer response. We hypothesized that combination of olaparib with cisplatin could enhance the efficacy of cisplatin in CC and help in improvement of platinum based therapy in CC. Our expression profile data of PARP1 demonstrates significantly increased expression in primary cervical cancer tissue biopsies and CC cell lines (Fig. 1). There are only two reports that have shown higher activity of PARP and its expression in CC compared to normal tissue in limited experiments but they have not provided any molecular mechanism of the contribution of PARP in CC cells^45,46^. Olaparib inhibits the PARylation of DNA bound PARP and its dissociation from the DNA strand and inhibits its enzymatic activity. Therefore, the presence of PARP molecule is critical for the action of PARP inhibitors which is more lethal than depletion of PARP protein^24,25,47^. Similar to earlier report that PARP hyperactivation is linked with cisplatin resistance^23^, our results show PARP hyperactivation and overexpression after the cisplatin treatment suggesting contribution of PARP in cisplatin resistance in CC. PAR polymers vary in length from 16 to >60 ADP-ribose polymers, depending upon the amount of genomic stress^48^. Overexpression of PARP in cisplatin treated cells also shows formation of smaller PAR polymers in early treatment which gradually increases with the increasing duration or the drug concentration and subsequently it allowed us to implicate the action of olaparib, i.e., trapping of more PARP molecules in cisplatin treated cells. Our data also demonstrate that olaparib was able to inhibit cisplatin induced PARylation and exert enhanced DNA damage in CC cells (Figs 2 and 3). Several groups have already reported that olaparib potentially drive cisplatin efficacy in the cancers with or without DNA repair defective pathways^12,49^. Interestingly, olaparib was able to increase cisplatin induced loss of clonogenic survival (Fig. 4). The disruption of Fanconi Anemia-BRCA (FA-BRCA) pathway due to promoter hyper-methylation of FANCF in CC has already been shown^50^. FANC genes potentially participate in DNA repair mechanism induced by cisplatin or alkylating agents as normal cellular response^51^. FANCF interacts with BRCA1 and other proteins to initiate other cellular responses in the cancer cells^51^. Another finding demonstrates that deficiency of FANC genes sensitizes cancer cells to PARP inhibitors^16^. The potential anti-proliferative effects of olaparib in combination with cisplatin demonstrated in our results may be possibly due to inactivation of FA-BRCA pathway in CC. While both PARP depleted and PARP inhibited cells show sensitivity to cisplatin, PARP inhibited cells displayed enhanced sensitivity. RPA loss reportedly sensitizes the cells to PARP inhibitors and several therapeutic agents and enhanced cell death^16,52^. In line with earlier reports, we observed that both PARP depleted and inhibited CC cells display loss of RPA foci due to lack of PARP activity which regulate the recruitment of RPA via MRE11. Here in CC cells, both inhibition and depletion of PARP show lack of PARP activity and subsequent loss of RPA foci formation after cisplatin treatment. Inhibition of proper RPA recruitment results in open DNA ends and formation of DSBs and persistent 53BP1 recruitment. Similarly, cisplatin treated PARP silenced cells or cells treated with combination of drugs show increased numbers of 53BP1 foci as compared to single drug. Collectively, our data suggest that PARP inhibition leads to increased DSBs in replication stressed cells by collapsing replication fork (Figs 5 and 6).

PARP potentially participates in smooth progression of cell cycle and potentially promotes G2/M arrest compared with G1 following genotoxic stress^53^ and cisplatin is also documented for inducing checkpoint kinases and cell cycle arrest^54,55^. Our data show that olaparib or cisplatin alone is able to induce G2/M phase arrest. Remarkably, olaparib treatment in combination with cisplatin displayed enhanced effect and displayed prolonged cell cycle arrest and enhanced DSBs in late S and G2/M phase (Fig. 7). PARP is well documented for its role in SSBR/BER in co-operation with XRCC1^56–58^, while some groups have also documented contradictory findings that PARP1 has no direct role in SSBR/BER pathway of DNA repair upon DNA damage^59,60^. Our results demonstrate that PARP inhibited and silenced CC cells show significant inhibition of XRCC1 recruitment at DNA damage sites. Data suggest that active PARP is required for completion of SSBR/BER. The reduction in XRCC1 foci in PARP inhibited cells results in accumulation of γH2A.X foci and subsequent conversion of SSB into double strand breaks (DSBs). PARP trapping potentially interferes with recruitment of several repair factors by creating steric hindrance around the DNA ends or by losing its enzymatic activity and loss of direct interaction with other proteins. Acetylation of PARP and other repair proteins displayed loss of PARP activity and it’s trapping at DNA ends^61^. A recent report also suggests that combination of PARP inhibitor with low dose of DNMT1 inhibitors increases PARP trapping at chromatin and induces enhanced cell death^62^. PARP displayed higher binding tendency to the DNA ends than KU70/80 and other damage sensors^28^. Our data suggest that PARP inhibition potentially decreases the relocation of KU80 and XRCC4 at damage ends by creating steric hindrance, whereas depletion of PARP has no such effect (Fig. 8). Trapping and silencing of PARP has no such effect on Rad51 recruitment as well. This data indicates that although PARP has no direct interaction with NHEJ effectors, its inhibition leads to the impairment of NHEJ pathway and enhanced DNA damage in CC cells. Production of more DSBs and failure of repair machinery in CC cells due to combined treatment of PARP inhibitor and cisplatin ultimately results in increased apoptosis and cell death (Fig. 9).

We also investigated the contribution of PARP in CC metastasis and its DNA repair independent functions. Previous studies have shown that PARP potentially regulates metastatic events like migration and invasion in other cancers^19–21^. Similarly, we have also demonstrated that olaparib treatment decreases vimentin expression in CC cells. We also demonstrate that sub-lethal dose of PARP inhibitor exerts significant anti-migratory and anti-invasive effect on CC cells and simultaneously decreases anchorage independent cell survival and induces apoptosis in CC cells by promoting anoikis as a single agent (Fig. 10).

Our study demonstrates that the disruption of PAR-γH2A.X association and loss of RPA results in formation of DSBs and persistent recruitment of 53BP1 in cervical cancer cells leading to subsequent cell cycle arrest and cell death. Inhibition of recruitment of broad spectrum DNA repair factors from different repair pathways i.e. BER and NHEJ contributes to failure of cisplatin induced DNA damage repair. Interestingly, our data also suggest the *in-vitro *anti-metastatic property of olaparib in cervical cancer cells, although it will need further *in-vivo* validation.

## Methods and Materials

### Cell culture and reagents

Human cervical cancer cell lines SiHa and ME180 were obtained from ATCC and maintained in DMEM (Gibco) supplemented with 10% fetal bovine serum (Gibco) and 5% humidified CO_2_. PARP inhibitor Olaparib (AZD2281) was purchased from Selleckchem, Houston, USA. Cisplatin was purchased from Bio Vision, CA, USA. Anti-PARP antibody [clone 46D11, #9532 (WB 1:1000 and IF 1:800)], anti-vimentin [clone D21H3, #5741 (WB 1:1000)] and cell lysis buffer were obtained from Cell Signaling Technology, MA, USA. Anti-PAR antibody [#4336-APC-050 (WB 1:1200 and IF 1:400)] was purchased from Trevigen, MD, USA. Anti-γH2A.X (p-Ser139) antibody [clone EP854(2)Y, #ab81299 (IF 1:2000)] was purchased from Abcam, Alexa flour 488 anti-γH2A.X (phospho-S139) [clone 2F3, #613405 (IF 1:400 and FACS 1:200)] antibody was purchased from Bio Legend, San Diego, California and anti-GAPDH antibody [clone 2D4A7, #NB300–328 (WB 1:3000)] was obtained from Imgenex. XRCC4 (IF 1:200) and RPA (IF 1:400) antibodies were gifted by Dr. A.S. Balajee, Columbia University, New York, USA. XRCC1 (IF 1:500), 53BP (IF 1:500), RAD51 (IF 1:100) and Ku80 (IF 1:400) antibodies were kind gift from Dr. Sathees C. Raghavan, Indian Institute of Science, Bangalore.

### Primary tumor biopsy and normal cervical tissue biopsy

Cervical cancer biopsies (n = 35; IIA (n = 3), IIB (n = 12), IIIB (n = 20) and normal cervical tissue (n = 15) (hysterectomy cases) were collected from Sir Sunderlal Hospital, Banaras Hindu University after written informed consent of the patients according to the approved protocol by institutional ethical committee of the Institute of Medical Sciences, Banaras Hindu University. Sample's inclusion or exclusion criteria were as described in Yadav *et al*.^63^. Briefly, normal cervical tissues were taken from the non-inflamed epithelial layer of ectocervix of patients undergoing hysterectomy due to either fibroid or prolapsed uterus. Ectocervix is the part of cervix which has squamous lining (glandular elements are present in the endocervix and at the squamocolumnar junction). Histology of normal samples and inflammation status was further confirmed by hematoxylin-eosin staining of tissue sections and samples having inflammation and glandular epithelium were excluded from the study. All the methods were performed in accordance with the relevant guidelines and regulations approved by institutional ethical committee.

### siRNA transfection

Cells were plated in 12 well plated in antibiotic free medium. 70–80% confluent cells were subjected to transfection. PARP siRNA and non-specific control siRNA were purchased from Santa Cruz Biotechnology. Transfection was carried out using OptiMEM^TM^ medium and Lipofectamine^TM^ 3000 regent (Invitrogen, CA, USA). Transfection was performed by mixing siRNA with Lipofectamine reagent in OptiMEM^TM^ medium and cells were incubated with the complex for 12 hrs. After the transfection, medium was replaced with normal growth medium and experiments were carried out as required.

### Cell viability assay

Cells were counted using Bio-Rad TC10 automated cell counter and 5 × 10^3^ cells were seeded in 96 well plate(s) in triplicates and allowed to adhere overnight. Cells were incubated with cisplatin and/or olaparib with different concentrations or time. After treatment, cells were incubated with 6 mg/ml MTT solution and plates were further incubated in CO_2_ incubator for 3 hrs. After incubation, formazan crystals were dissolved in DMSO and plate(s) were scanned with BioRad iMark Microplate reader at 570 nm.

### Clonogenic survival assay

Clonogenic survival was performed as previously describe^64^. Briefly CC cells were seeded in 6 well plates in very low density and allowed to adhere overnight. Next day cells were treated with drugs as indicated for 24 hrs. After 24 hrs, drugs were washed out and cells were grown in fresh growth medium for next 2 weeks. After formation of proper colonies (at least 50 cells/colony) in untreated cells, colonies were fixed into 4% paraformaldehyde and stained with 0.5% crystal violet and counted.

### Protein extraction and western blot

Total protein was isolated from tissue and cell line using cell lysis buffer and protein quantity was determined via Bradford reagent. Equal amounts of protein was separated on SDS-PAGE and transferred to PVDF membrane. Membrane was then blocked with 5% non-fat milk in TBST for 2 hrs at room temperature (RT) and then hybridized overnight with specific primary antibody. After primary antibody incubation, membrane was washed 3 times with TBST and HRP labeled secondary antibody was added for 2 hrs at RT. Signal was detected using chemi-luminiscence on X-ray film.

### Immunohistochemistry (IHC)

Immunohistochemistry was done on paraffin embedded tissue sections which were mounted on poly L-lysine coated slides. Tissue sections were deparaffinized, dehydrated and treated for 10 minutes in citrate buffer for antigen retrieval in water bath at 95 °C. Sections were incubated in primary antibody diluted in blocking solution overnight at 4 °C. After incubation peroxidase conjugated secondary antibody was used against the primary antibody. For chromogenic detection, 3,3′-diaminobenzidine tetrahydrochloride (DAB) (Sigma, USA) was used as the substrate for peroxidase. Slides were counterstained with hematoxylin. Cells with brown nuclei were considered as positively stained for PARP1. Negative control experiment was performed without using primary antibody.

### Immunofluoresence

Cells were grown on cover slips in 12 well plates. Drug treatment was done as indicated in result sections. Upon completion of the treatments the cells were fixed in chilled methanol and blocked with blocking solution (1% BSA + 5% normal goat serum + 0.3M glycine and 0.01% Titron-X100) for 30 min at RT. Cells were then incubated with specific primary antibody or only with secondary antibody (isotype control) diluted in blocking solution and incubation was done in humid chamber at 4 °C overnight. After incubation, coverslip was washed 3 times with PBS and FITC or TRITC labeled secondary antibody was added for 2 hrs at RT. Cells were then washed and nucleus was stained with DAPI. Cell with more than 20 foci/cells were taken as positive for signal. Slides were scanned under scanning confocal microscope LMS780.

### Cell cycle analysis

Cells were treated with cisplatin and/or olaparib as indicated in result section. After treatment, the cells were fixed with 70% chilled methanol for 2 hours at −20 °C. Cells were then washed with PSB and further incubated with RNaseA (250 μg/ml) in PBS for 2 hr at 37 °C and then stained with PI (1 mg/ml). Stained cells were analyzed using BD FACS caliber and analysis was done using Cell Quest Pro software.

### Apoptosis assay

Cells were grown in 6 well plates and treated with cisplatin and/or olaparib. After completion of the treatment the cells were harvested and washed with PBS 2 times. After washing cell were re-suspended in 200 µl 1× Annexin binding buffer containing PI and anti-Annexin-FITC antibody and cells were incubated at RT for 15 min in dark. After incubation stained cells were analyzed by BD FACS caliber in FL1 and FL2 channels. Data was analyzed using Cell Quest Pro software.

### Anoikis assay

Multi-well plates were coated with Poly-Hema and cells were cultured in suspension condition as indicated in result section. After culture and drug treatment, cells were centrifuged and washed with PBS 2 times on ice. After washing, the cells were stained with PI (1mg/ml) for 15 min at RT and cells were analyzed by BD FACS caliber.

### Transwell migration assay

Overnight serum starved cells were trypsinized and 20,000 cells were seeded in incomplete medium with or without drug in upper chamber of transwell insert. Insert was then placed in 24 well plates and lower part of the well was filled with 750 µl of medium supplemented with 10% FBS as chemo-attractant. The setup was then incubated in incubator for indicated duration. After incubation non migrated cells (cells in upper chamber) were removed by swapping with cotton swap and migrated cells were fixed with 4% paraformaldehyde, permeabilized with 100% methanol and stained with 0.5% crystal violet. After staining porous membrane was excised out and mounted on slide and migrated cells were photographed on Nikon 80*i* microscope. Several random fields were imaged and cells were counted in three independent experiments.

### Transwell invasion assay

Invasion assay was performed similarly as migration assay except coating the insert with Basement Membrane Matrix (BMM) or Matrigel. Briefly, transwell insert was coated with BMM and allowed to solidify at 37 °C for 1 hr prior to cell seeding. 20,000 cells were then seeded on the top of the BMM with or without drug and cells were allowed to invade in BMM for indicated duration. After incubation for desired duration non-invaded cells were wiped out and invaded cells were fixed, stained and counted as described in migration assay.

### Statistical analysis

All experiments were repeated three times until stated. Statistical analysis was done using Graph pad prism software Ver. 5.0.

## Electronic supplementary material

Supplementary Information

## References

[1] D’Amours D, Desnoyers S, D’Silva I, Poirier GG (1999). Poly(ADP-ribosyl)ation reactions in the regulation of nuclear functions. The Biochemical journal.

[2] Ossovskaya V (2010). Upregulation of Poly (ADP-Ribose) Polymerase-1 (PARP1) in Triple-Negative Breast Cancer and Other Primary Human Tumor Types. Genes & cancer.

[3] Ba X, Garg NJ (2011). Signaling mechanism of poly(ADP-ribose) polymerase-1 (PARP-1) in inflammatory diseases. The American journal of pathology.

[4] Scott CL, Swisher EM, Kaufmann SH (2015). Poly (ADP-ribose) polymerase inhibitors: recent advances and future development. Journal of clinical oncology: official journal of the American Society of Clinical Oncology.

[5] Audeh MW (2010). Oral poly(ADP-ribose) polymerase inhibitor olaparib in patients with BRCA1 or BRCA2 mutations and recurrent ovarian cancer: a proof-of-concept trial. Lancet.

[6] Ledermann J (2012). Olaparib maintenance therapy in platinum-sensitive relapsed ovarian cancer. The New England journal of medicine.

[7] Liu JF (2013). A Phase 1 trial of the poly(ADP-ribose) polymerase inhibitor olaparib (AZD2281) in combination with the anti-angiogenic cediranib (AZD2171) in recurrent epithelial ovarian or triple-negative breast cancer. European journal of cancer.

[8] Tutt A (2010). Oral poly(ADP-ribose) polymerase inhibitor olaparib in patients with BRCA1 or BRCA2 mutations and advanced breast cancer: a proof-of-concept trial. Lancet.

[9] Dent RA (2013). Phase I trial of the oral PARP inhibitor olaparib in combination with paclitaxel for first- or second-line treatment of patients with metastatic triple-negative breast cancer. Breast cancer research: BCR.

[10] Bryant HE (2005). Specific killing of BRCA2-deficient tumours with inhibitors of poly(ADP-ribose) polymerase. Nature.

[11] Cheng H (2013). PARP inhibition selectively increases sensitivity to cisplatin in ERCC1-low non-small cell lung cancer cells. Carcinogenesis.

[12] Michels J (2013). Synergistic interaction between cisplatin and PARP inhibitors in non-small cell lung cancer. Cell cycle.

[13] Postel-Vinay S (2013). A high-throughput screen identifies PARP1/2 inhibitors as a potential therapy for ERCC1-deficient non-small cell lung cancer. Oncogene.

[14] Gilardini Montani MS (2013). *ATM-depletion in breast cancer cells confers sensitivity* to PARP inhibition. Journal of experimental & clinical cancer research.

[15] Koppensteiner R (2014). Effect of MRE11 loss on PARP-inhibitor sensitivity in endometrial cancer *in vitro*. PloS one.

[16] McCabe N (2006). Deficiency in the repair of DNA damage by homologous recombination and sensitivity to poly(ADP-ribose) polymerase inhibition. Cancer research.

[17] Patel AG, Sarkaria JN, Kaufmann SH (2011). Nonhomologous end joining drives poly(ADP-ribose) polymerase (PARP) inhibitor lethality in homologous recombination-deficient cells. Proceedings of the National Academy of Sciences of the United States of America.

[18] Passaro C (2015). PARP inhibitor olaparib increases the oncolytic activity of dl922–947 in *in vitro* and *in vivo* model of anaplastic thyroid carcinoma. Molecular oncology.

[19] Choi EB (2016). PARP1 enhances lung adenocarcinoma metastasis by novel mechanisms independent of DNA repair. Oncogene.

[20] Chu S, Xu H, Ferro TJ, Rivera PX (2007). Poly(ADP-ribose) polymerase-1 regulates vimentin expression in lung cancer cells. *American journal of physiology*. Lung cellular and molecular physiology.

[21] Rodriguez MI (2013). PARP-1 regulates metastatic melanoma through modulation of vimentin-induced malignant transformation. PLoS genetics.

[22] Thaker PH (2017). A phase I trial of paclitaxel, cisplatin, and veliparib in the treatment of persistent or recurrent carcinoma of the cervix: an NRG Oncology Study (NCT#01281852). Annals of oncology: official journal of the European Society for Medical Oncology.

[23] Michels J (2013). Cisplatin resistance associated with PARP hyperactivation. Cancer research.

[24] Murai J (2012). Trapping of PARP1 and PARP2 by Clinical PARP Inhibitors. Cancer research.

[25] Godon C (2008). PARP inhibition versus PARP-1 silencing: different outcomes in terms of single-strand break repair and radiation susceptibility. Nucleic acids research.

[26] Mansour WY, Rhein T, Dahm-Daphi J (2010). The alternative end-joining pathway for repair of DNA double-strand breaks requires PARP1 but is not dependent upon microhomologies. Nucleic acids research.

[27] Schultz N (2003). Nucleic acids research.

[28] Wang M (2006). PARP-1 and Ku compete for repair of DNA double strand breaks by distinct NHEJ pathways. Nucleic acids research.

[29] Campalans A (2013). Distinct spatiotemporal patterns and PARP dependence of XRCC1 recruitment to single-strand break and base excision repair. Nucleic acids research.

[30] El-Khamisy SF (2003). A requirement for PARP-1 for the assembly or stability of XRCC1 nuclear foci at sites of oxidative DNA damage. Nucleic acids research.

[31] Das M (2015). HPV-type-specific response of cervical cancer cells to cisplatin after silencing replication licensing factor MCM4. Tumour biology: the journal of the International Society for Oncodevelopmental Biology and Medicine.

[32] Wagner JM, Karnitz LM (2009). Cisplatin-induced DNA damage activates replication checkpoint signaling components that differentially affect tumor cell survival. Molecular pharmacology.

[33] Cruet-Hennequart S (2009). Characterization of the effects of cisplatin and carboplatin on cell cycle progression and DNA damage response activation in DNA polymerase eta-deficient human cells. Cell cycle.

[34] Beskow C (2009). Radioresistant cervical cancer shows upregulation of the NHEJ proteins DNA-PKcs, Ku70 and Ku86. British journal of cancer.

[35] Bouwman P, Jonkers J (2012). The effects of deregulated DNA damage signalling on cancer chemotherapy response and resistance. *Nature reviews*. Cancer.

[36] Brozovic A, Osmak M (2007). Activation of mitogen-activated protein kinases by cisplatin and their role in cisplatin-resistance. Cancer letters.

[37] Galluzzi L (2012). Molecular mechanisms of cisplatin resistance. Oncogene.

[38] Huang EY (2012). *A novel radioresistant* mechanism of galectin-1 mediated by H-Ras-dependent pathways in cervical cancer cells. Cell death & disease.

[39] Kwok JM (2010). FOXM1 confers acquired cisplatin resistance in breast cancer cells. Molecular cancer research:.

[40] Martin LP, Hamilton TC, Schilder RJ (2008). Platinum resistance: the role of DNA repair pathways. Clinical cancer research: an official journal of the American Association for Cancer Research.

[41] Mehta, F. F., Baik, S. & Chung, S. H. Recurrence of cervical cancer and its resistance to progestin therapy in a mouse model. *Oncotarget*, 10.18632/oncotarget.13676 (2016). 10.18632/oncotarget.13676 PMC5356807 27911853

[42] Frankum J (2015). Complementary genetic screens identify the E3 ubiquitin ligase CBLC, as a modifier of PARP inhibitor sensitivity. Oncotarget.

[43] Oplustilova L (2012). Evaluation of candidate biomarkers to predict cancer cell sensitivity or resistance to PARP-1 inhibitor treatment. Cell cycle.

[44] Turner NC (2008). A synthetic lethal siRNA screen identifying genes mediating sensitivity to a PARP inhibitor. The EMBO journal.

[45] Fukushima M (1981). Poly(ADP-ribose) synthesis in human cervical cancer cell-diagnostic cytological usefulness. Cancer letters.

[46] Hassumi-Fukasawa MK (2012). Expression of BAG-1 and PARP-1 in precursor lesions and invasive cervical cancer associated with human papillomavirus (HPV). Pathology oncology research: POR.

[47] Domagala, P., Lubinski, J. & Domagala, W. Iniparib in metastatic triple-negative breast cancer. *The New England journal ofmedicine* **364**, 1780; author reply 1781, 10.1056/NEJMc1101855#SA1 (2011). 10.1056/NEJMc1101855 21542760

[48] Andrabi SA (2006). Poly(ADP-ribose) (PAR) polymer is a death signal. Proceedings of the National Academy of Sciences of the United States of America.

[49] Shunkwiler L, Ferris G, Kunos C (2013). Inhibition of Poly(ADP-Ribose) Polymerase Enhances Radiochemosensitivity in Cancers Proficient in DNA Double-Strand Break Repair. International journal of molecular sciences.

[50] Narayan G (2004). Promoter hypermethylation of FANCF: disruption of Fanconi Anemia-BRCA pathway in cervical cancer. Cancer research.

[51] D’Andrea AD, Grompe M (2003). The Fanconi anaemia/BRCA pathway. *Nature reviews*. Cancer.

[52] Balajee AS, Geard CR (2004). Replication protein A and gamma-H2AX foci assembly is triggered by cellular response to DNA double-strand breaks. Experimental cell research.

[53] Masutani M, Nozaki T, Wakabayashi K, Sugimura T (1995). Role of poly(ADP-ribose) polymerase in cell-cycle checkpoint mechanisms following gamma-irradiation. Biochimie.

[54] Dasika GK (1999). DNA damage-induced cell cycle checkpoints and DNA strand break repair in development and tumorigenesis. Oncogene.

[55] Roos WP, Kaina B (2013). DNA damage-induced cell death: from specific DNA lesions to the DNA damage response and apoptosis. Cancer letters.

[56] Fisher AE, Hochegger H, Takeda S, Caldecott KW (2007). Poly(ADP-ribose) polymerase 1 accelerates single-strand break repair in concert with poly(ADP-ribose) glycohydrolase. Molecular and cellular biology.

[57] Javle M, Curtin NJ (2011). The role of PARP in DNA repair and its therapeutic exploitation. British journal of cancer.

[58] Orta ML (2014). The PARP inhibitor Olaparib disrupts base excision repair of 5-aza-2′-deoxycytidine lesions. Nucleic acids research.

[59] Strom CE (2011). Poly (ADP-ribose) polymerase (PARP) is not involved in base excision repair but PARP inhibition traps a single-strand intermediate. Nucleic acids research.

[60] Vodenicharov MD (2000). Base excision repair is efficient in cells lacking poly(ADP-ribose) polymerase 1. Nucleic acids research.

[61] Robert C (2016). Histone deacetylase inhibitors decrease NHEJ both by acetylation of repair factors and trapping of PARP1 at DNA double-strand breaks in chromatin. Leukemia research.

[62] Muvarak NE (2016). Enhancing the Cytotoxic Effects of PARP Inhibitors with DNA Demethylating Agents - A Potential Therapy for Cancer. Cancer cell.

[63] Yadav SS (2014). Epigenetic silencing of CXCR4 promotes loss of cell adhesion in cervical cancer. BioMed research international.

[64] Franken NA, Rodermond HM, Stap J, Haveman J, van Bree C (2006). Clonogenic assay of cells *in vitro*. Nature protocols.

