# Supplementary material for: Olaparib modulates DNA repair efficiency, sensitizes cervical cancer cells to cisplatin and exhibits anti-metastatic property
Source: Sci Rep. 2017 Oct 9;7:12876. doi: 10.1038/s41598-017-13232-3 (PMC5634505; doi:10.1038/s41598-017-13232-3)

Olaparib modulates DNA repair efficiency, sensitizes cervical cancer cells to cisplatin and exhibits anti-metastatic property

Chandra Bhushan Prasad[1], Shyam Babu Prasad[1], Suresh Singh Yadav[1], Laxmi Kant Pandey[2], Sunita Singh[3], Satyajit Pradhan[4], Gopeshwar Narayan[1]

Corresponding Author
Dr. Gopeshwar Narayan
Professor
Molecular and Human Genetics, Institute of Science, Banaras Hindu University
Varanasi-221005
gnarayan@bhu.ac.in

Supplementary figures S1-S12

Figure S1: Effect of Cisplatin and/or olaparib on HEK239 proliferation.

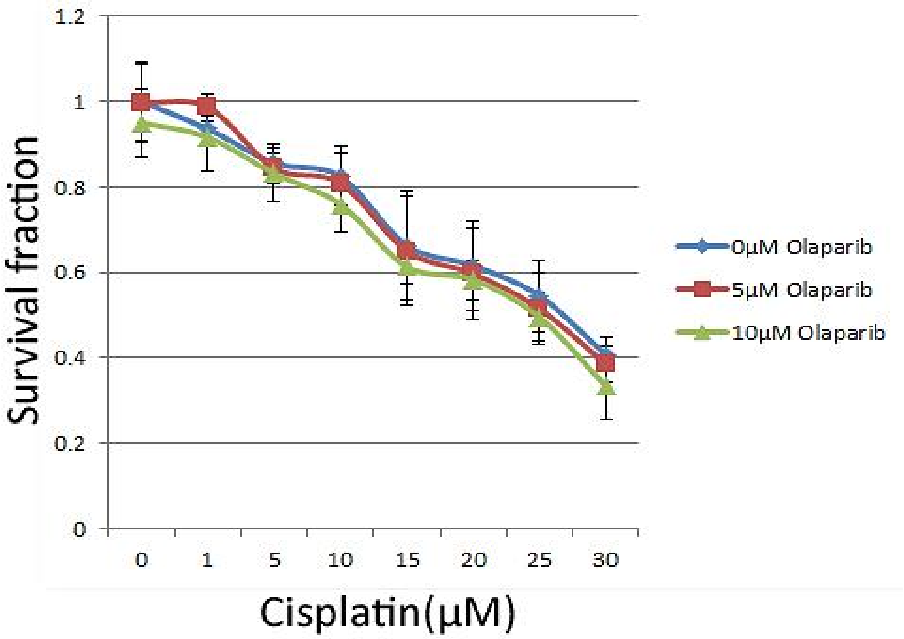

Figure S2. CC cell lines SiHa and ME180 were grown in 12 well plates on coverslip and treated with 10μM of cisplatin and/or olaparib for 24 hours. After 24 hrs drug media was replaced (DR) with fresh medium and cells were fixed immediately at 0 hr post drug removal (DR0). Further cells were grown and fixed at every 24 hrs till 10 days (DR0 to DR10). Fixed cells were stained with anti-γH2A.X antibody and counterstained with DAPI. Counting of γH2A.X foci in each group was done in blind fashion in 60× magnification in several fields. At least 200 cells were scored and frequency & distribution of γH2A.X foci were observed in both cell lines in each treatment group.

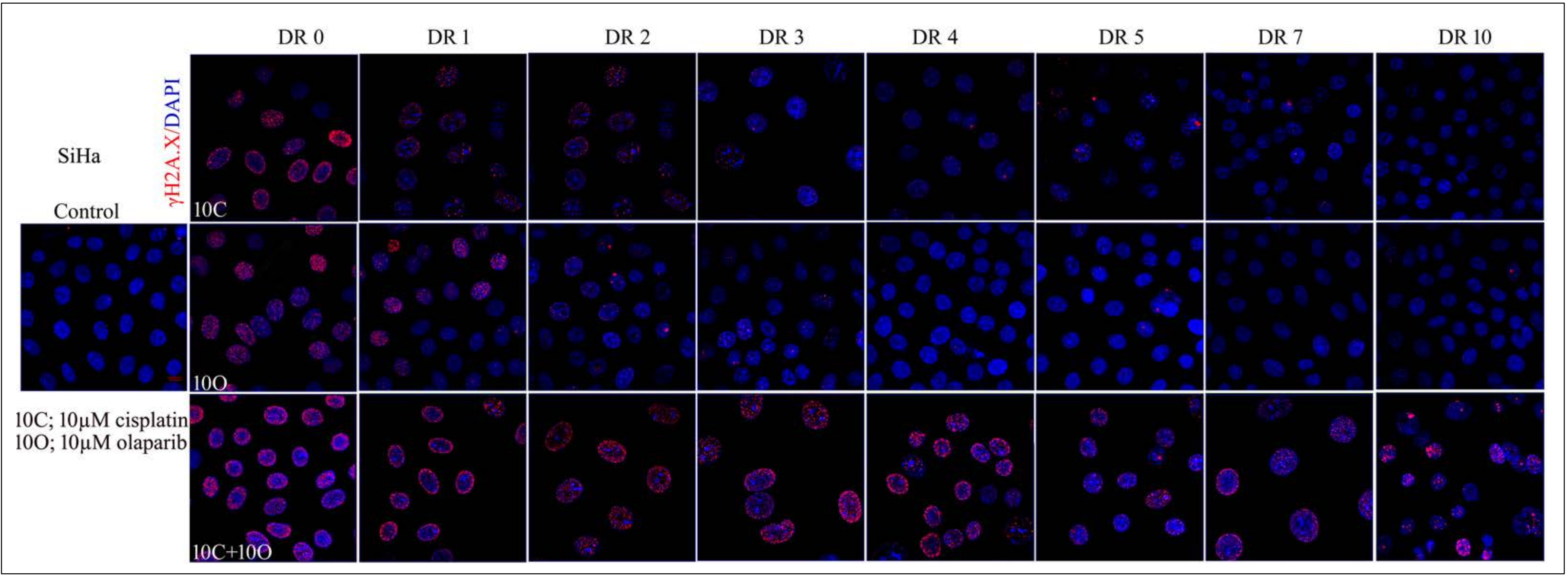

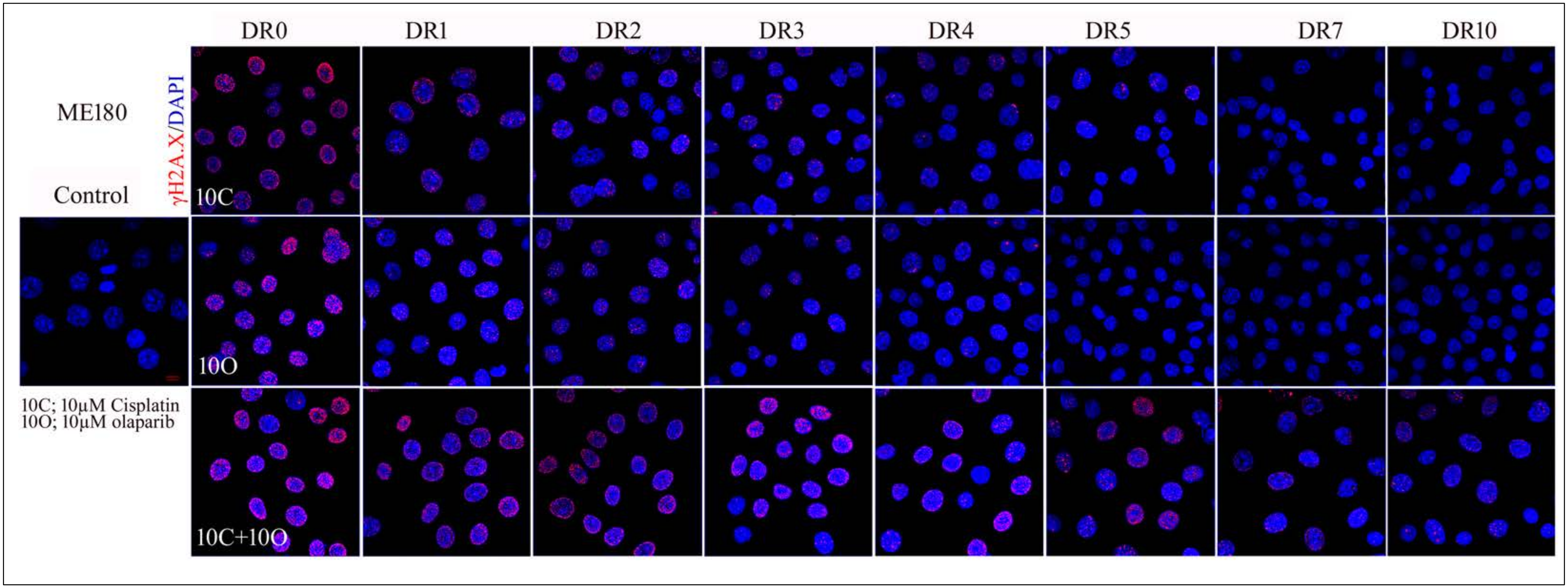
DR0
DR1
DR2
DR3
DR4
DR5
DR7
DR10
ME180
Control
γH2A.X/DAPI
10C
10O
10C+10O
10C; 10μM Cisplatin
10O; 10μM olaparib

Figure S3. Both the cell lines were treated as explained in previous experiments and cells were stained with FITC γH2A.X antibody and PI. Cells were analyzed by flow cytometer for γH2A.X signal. Cells were identified in cell cycle phases on linear scale on PI (FL2 channel) and γH2A.X signal was recorded in log scale on FITC channel (FL1).Total cells with positive γH2A.X signal (R1) were identified and gated in respect to isotype control (only secondary antibody). Further total γH2A.X cells were sub divided in G1 (Green), S (Magenta) and G2/M (Blue) on linear scale on PI. Cells positive for γH2A.X signals in each phase were analyzed and experiment was repeated two times independently.

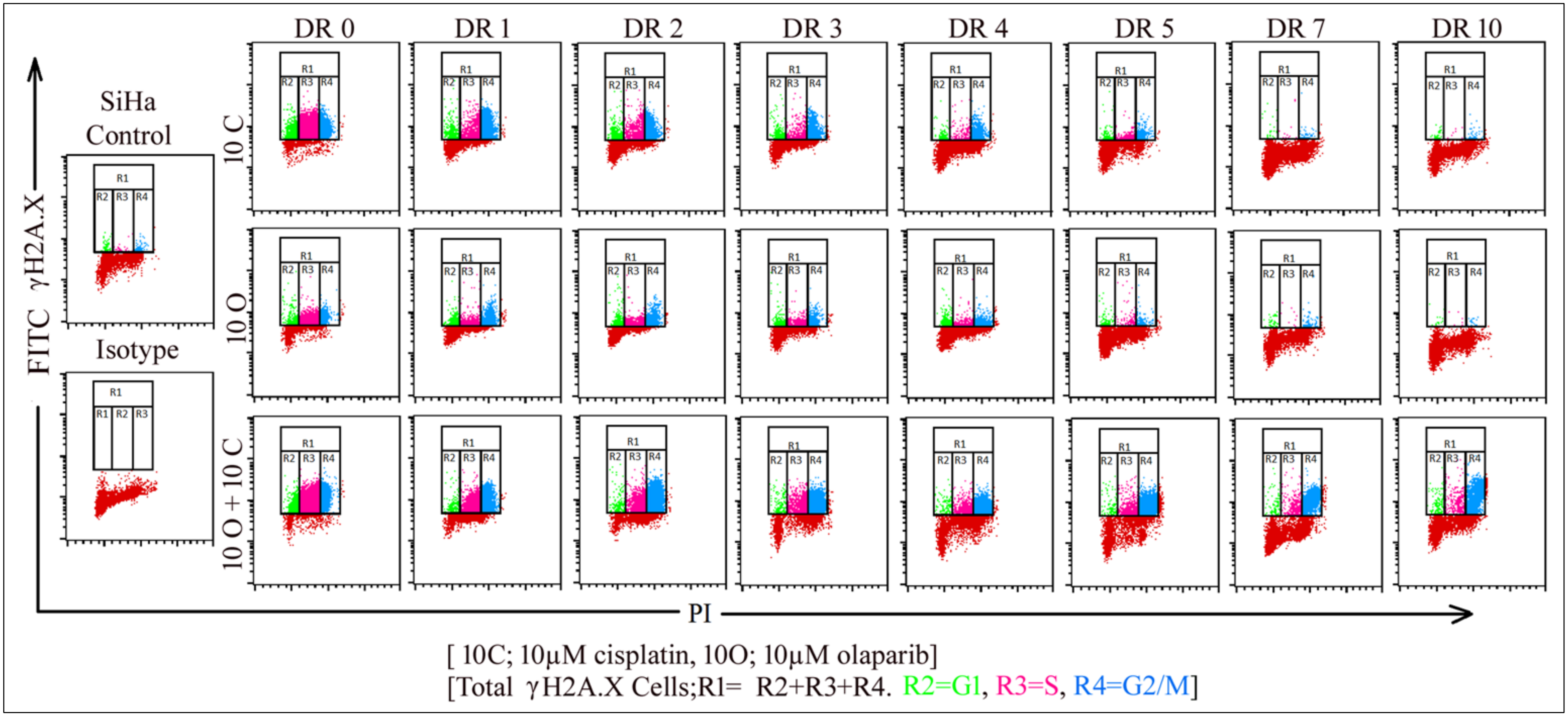

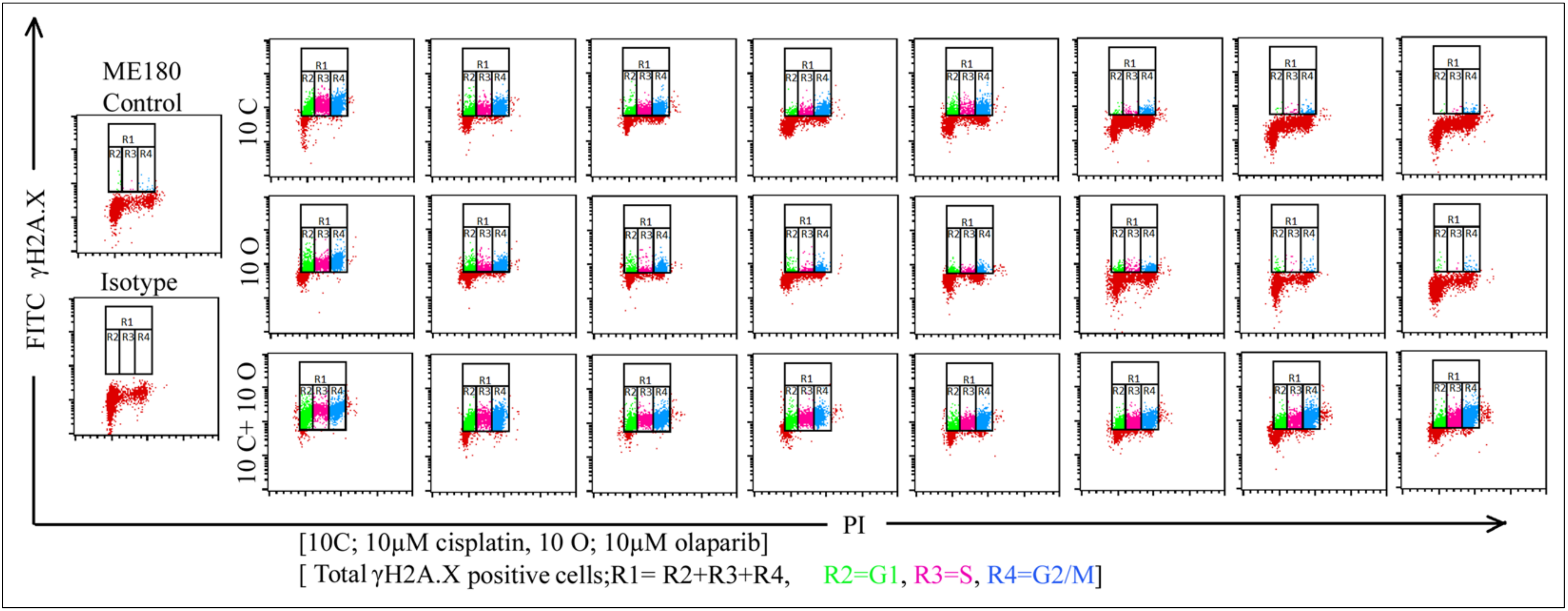
FITC γH2A.X
ME180
Control
Isotype
10 C
10 O
10 C+ 10 O
R1
R2
R3
R4
PI
[10C; 10μM cisplatin, 10 O; 10μM olaparib]
[Total γH2A.X positive cells;R1= R2+R3+R4, R2=G1, R3=S, R4=G2/M]

Figure S4. PARP siRNA transfected cells and scrambled siRNA transfected cells were treated with 5μM cisplatin for 3 hrs and drug was replaced with fresh growth medium. After media change cells were fixed at indicated time points (0, 3, 6, 12, 16, 24,36 and 48 hr) and γH2A.X foci were counted in blind fashion in two independent experiments in both the cell lines.

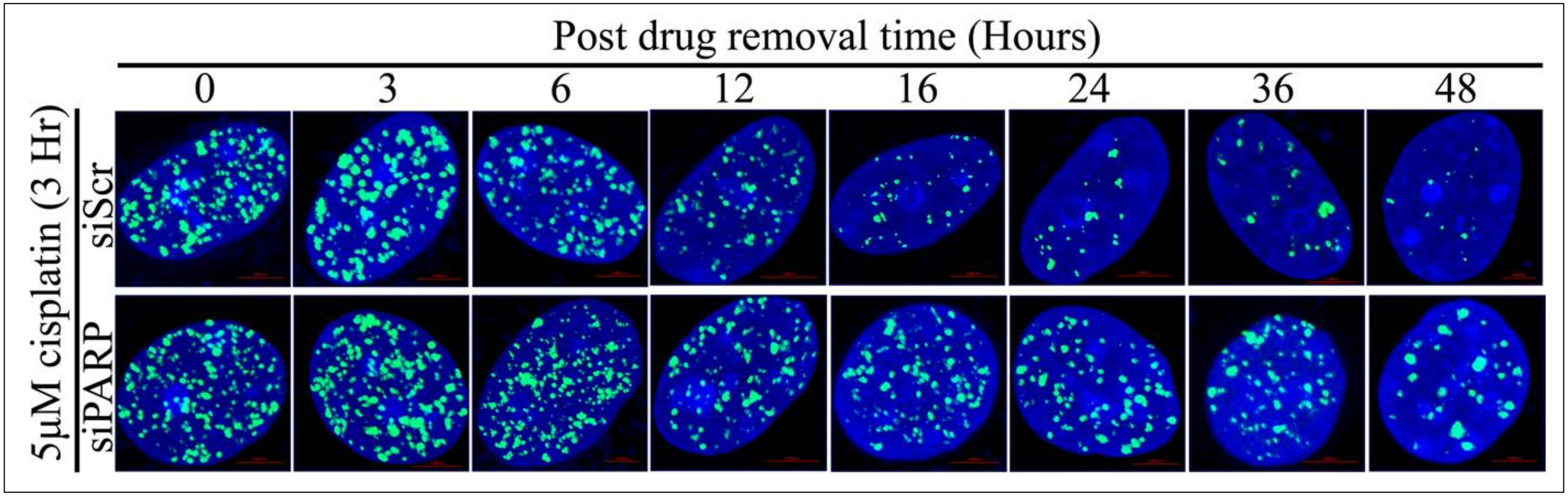

Figure S5. Both the cell lines were treated with 10μM of both cisplatin and/or olaparib. After drug treatment cells were fixed in chilled methanol at indicated time points (DR0 to DR10) and stained with PI and RNase A solution for 1hr at 37°C. Cell cycle analysis was performed and cell distribution in G1, S and G2/M phase was analyzed in each cell line in all treatment groups.

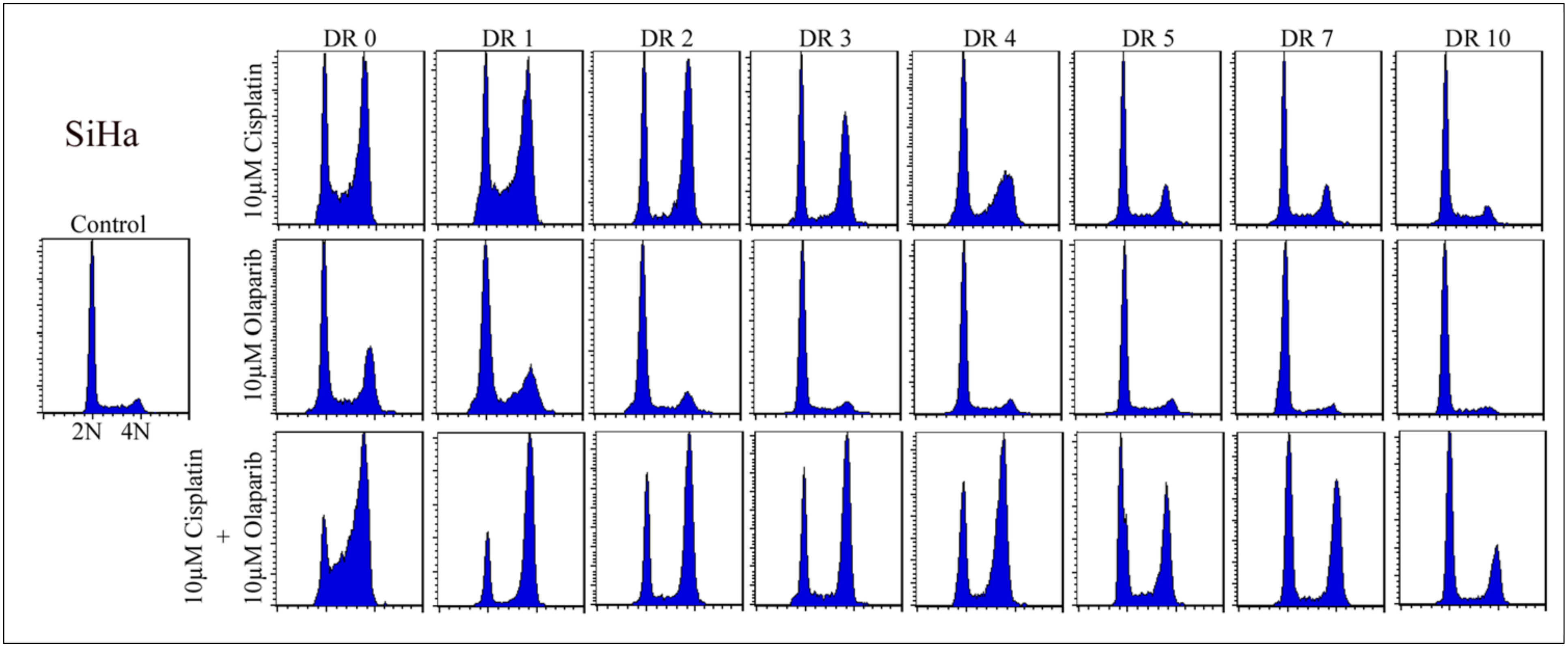

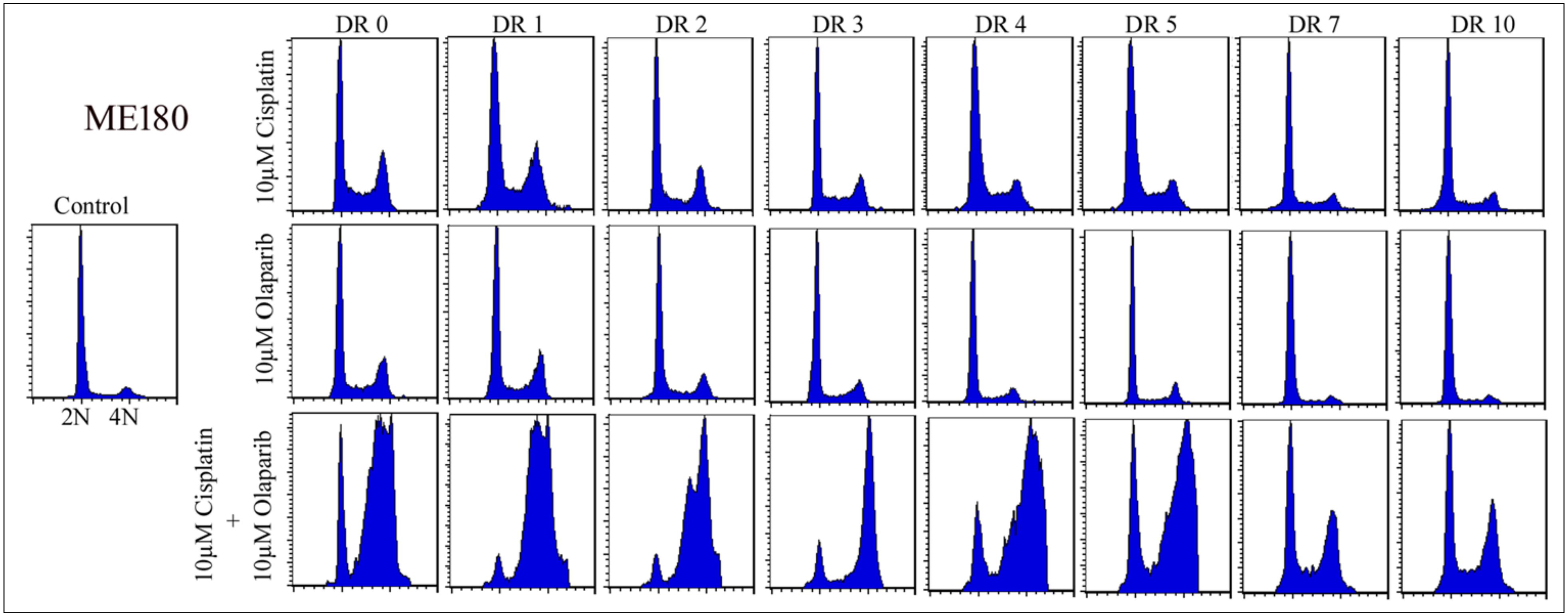

ME180
Control
2N
4N
DR 0
DR 1
DR 2
DR 3
DR 4
DR 5
DR 7
DR 10
10μM Cisplatin
10μM Olaparib
10μM Cisplatin
+
10μM Olaparib

Figure S6: Co-immunofluorescence of γH2A.X with Rad51. Cells were treated as indicated and co-staining of Rad51 and γH2A.X was carried out after 24hr treatment. Cell treated with combination of both drugs or cisplatin treated PARP silenced cells show no change on Rad51 recruitment at DSBs.

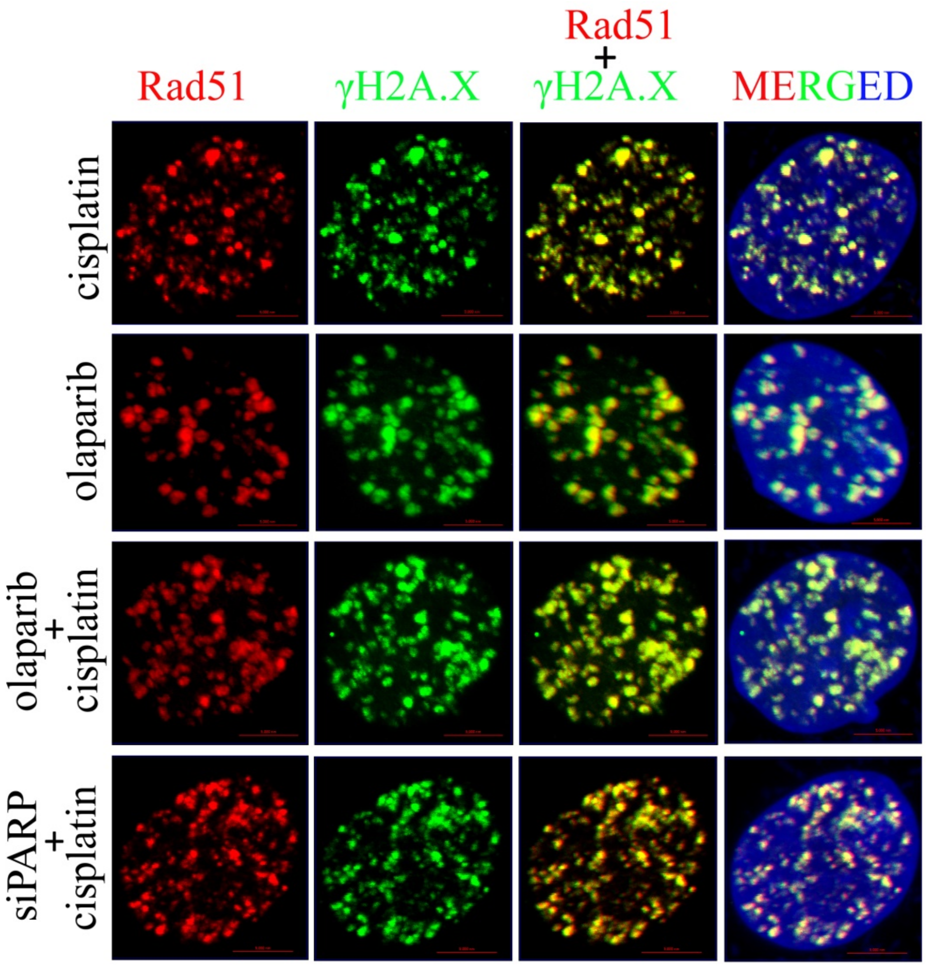

FigureS7. Both the CC cell lines were treated for 24 hrs as indicated and cells were stained with Annexin V/PI according to the manufacturer's protocol. Stained cells were analyzed in flow cytometer and Annexin V positive apoptotic cells (FL1 Chanel) and PI positive dead cells (FL2 Chanel) were scored.

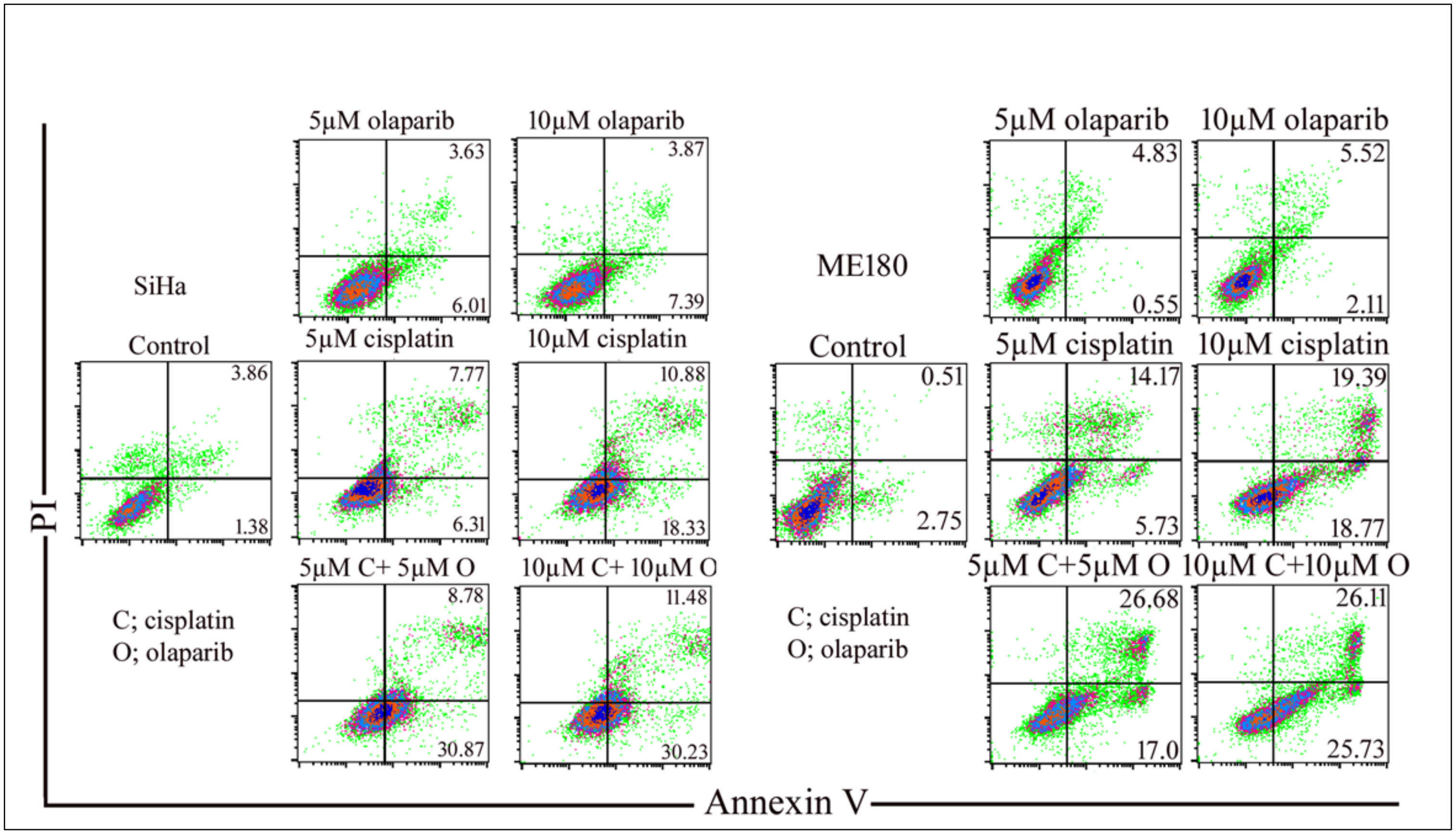

Figure S8. Both the CC cell lines were transfected with PARP siRNA and scrambled siRNA. Both transfected cells were treated for 24 hrs as indicated and cells were stained with Annexin V/PI according to the manufacturer's protocol. Stained cells were analyzed in flow cytometer and Annexin V positive apoptotic cells (FL1 Chanel) and PI positive dead cells (FL2 Chanel) were scored.

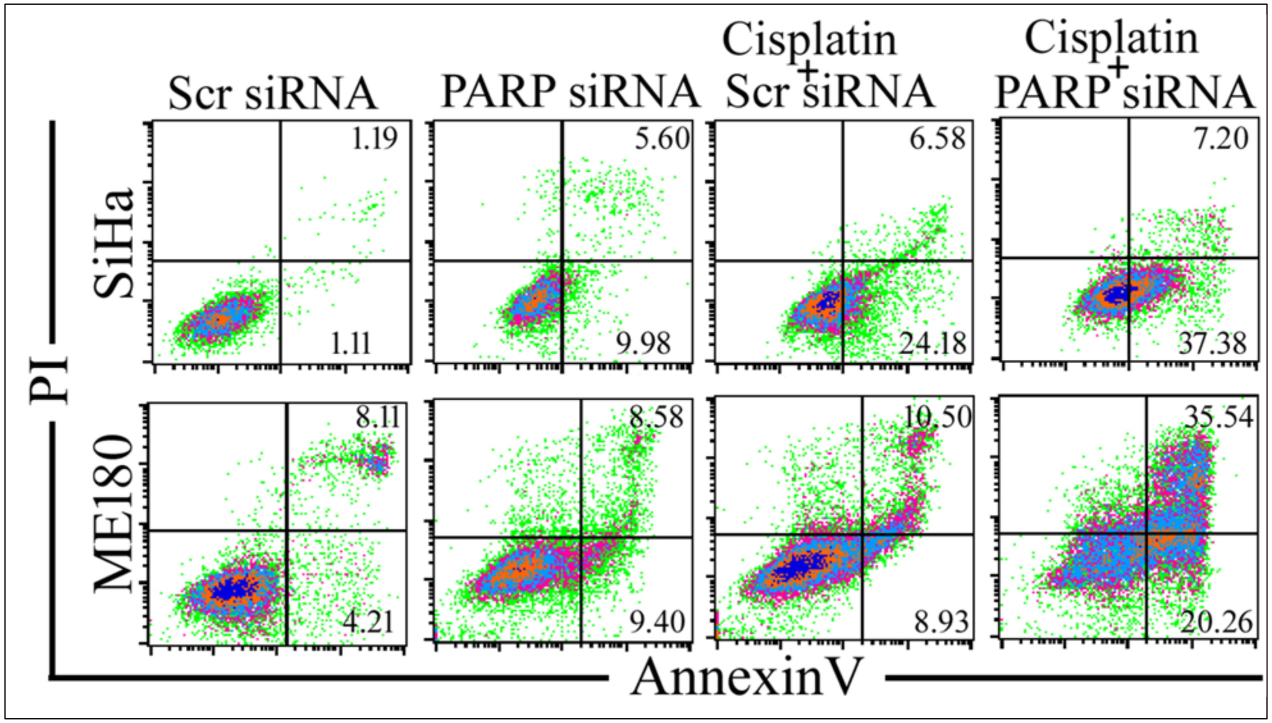

Figure S9: Cell were pre-treated with 10μM olaparib for 12 hrs and after that cells were stained with CFSE and migration and invasion was performed as described in method section. Olaparib treated cell display reduced migration and invasion. Similarly SiHa cell were tread with 10μM olaparib for 24hrs and total protein was subjected to western blot. Olaparib treated SiHa cell shows decrease in vimentin level. B-actin was loaded as internal control.

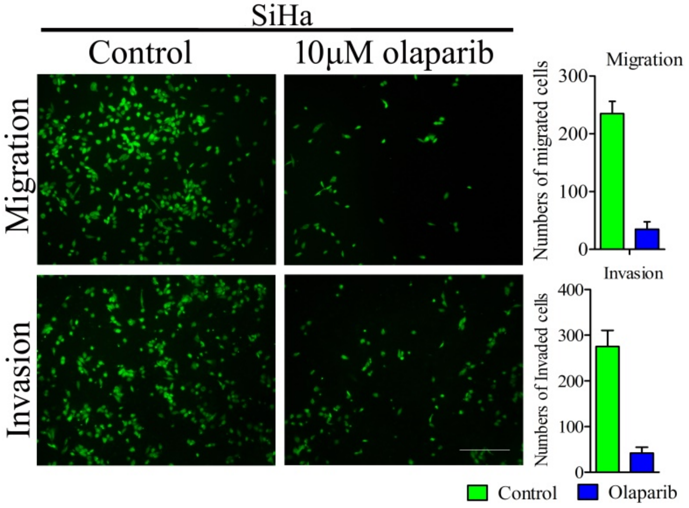

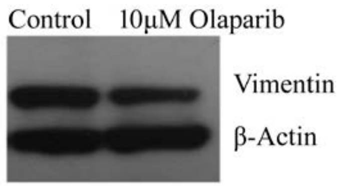

Figure S10. Both the CC cell lines were treated for 24 hrs as indicated in anchorage dependent (adherent) and anchorage independent (suspension) conditions. Total cells from all treatment groups were washed with 1X PBS and cells were stained with PI. A negative control was run to identify PI negative and PI positive area (gated area) and cells positive for PI signal (dead cells) were scored in all groups.

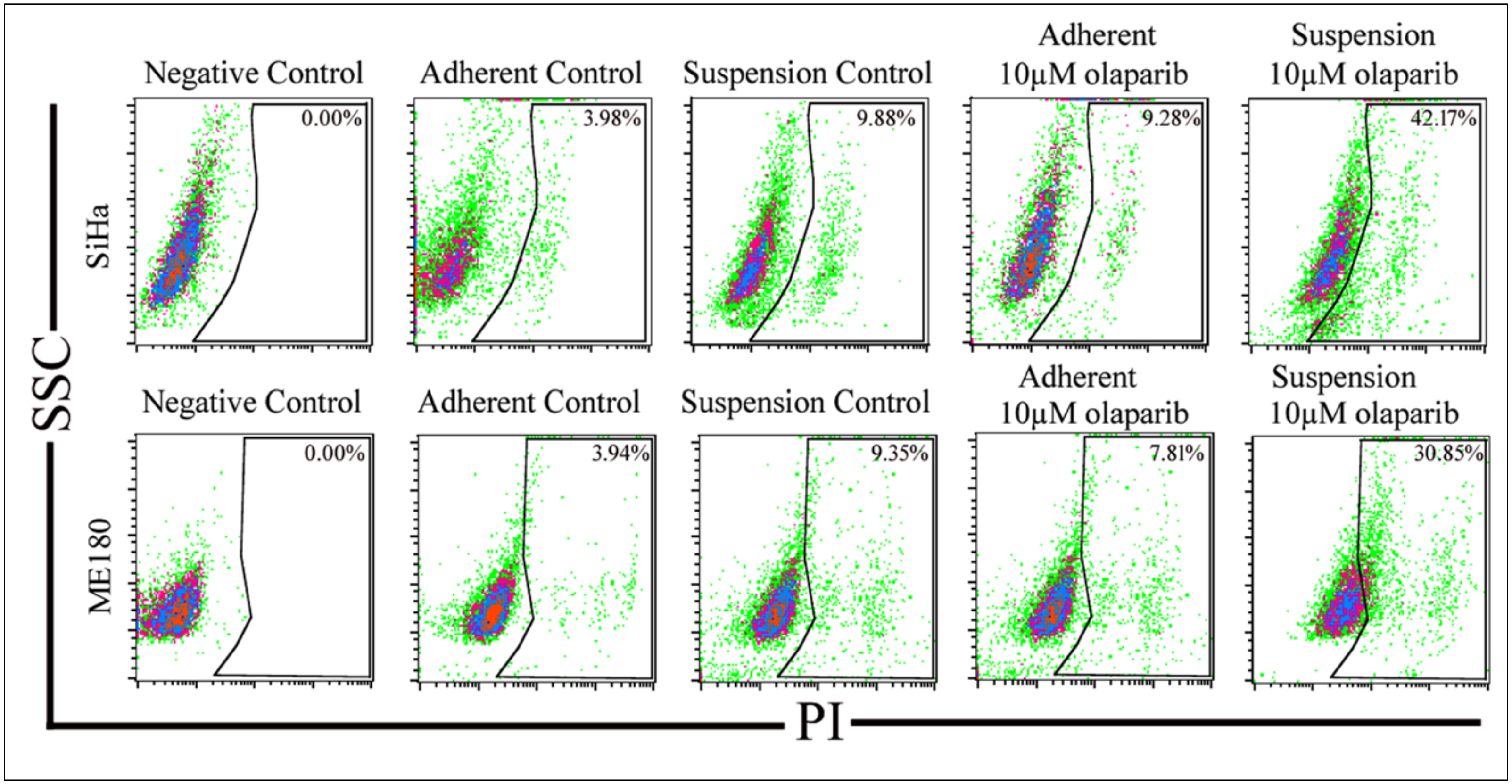

Figure S11. Both the CC cell lines were treated for 24 hrs with 10μM olaparib in anchorage dependent (adherent) and anchorage independent (suspension) conditions. Cells were stained with Annexin V/PI according to the manufacturer's protocol. Stained cells were analyzed in flow cytometer and Annexin V positive apoptotic cells (FL1 Chanel) and PI positive dead cells (FL2 Chanel) were scored.

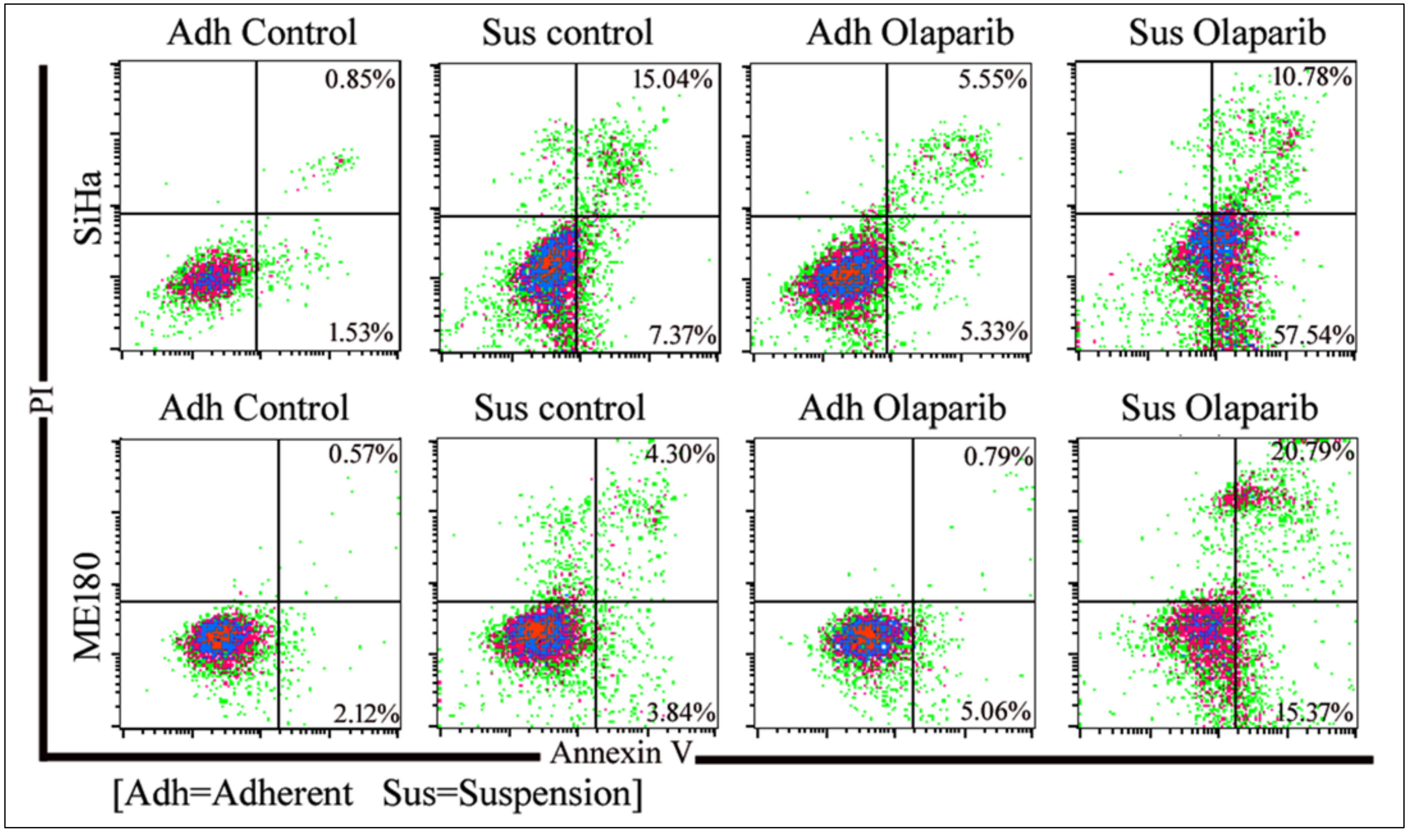

Figure S12. Non-cropped blot images. Rectangular lining indicating cropped area.

Full blot for Figure 1.

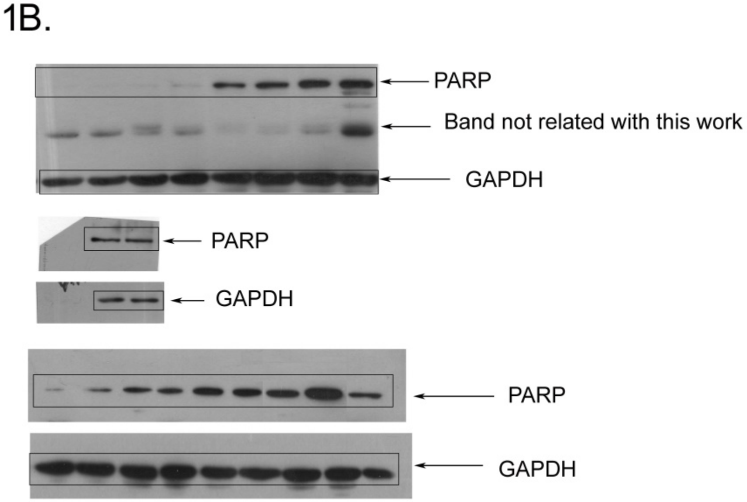

Full bot for figure 3.

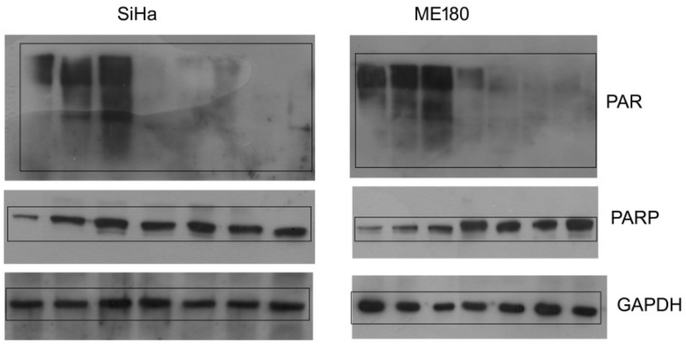

Full blot for figure 2.

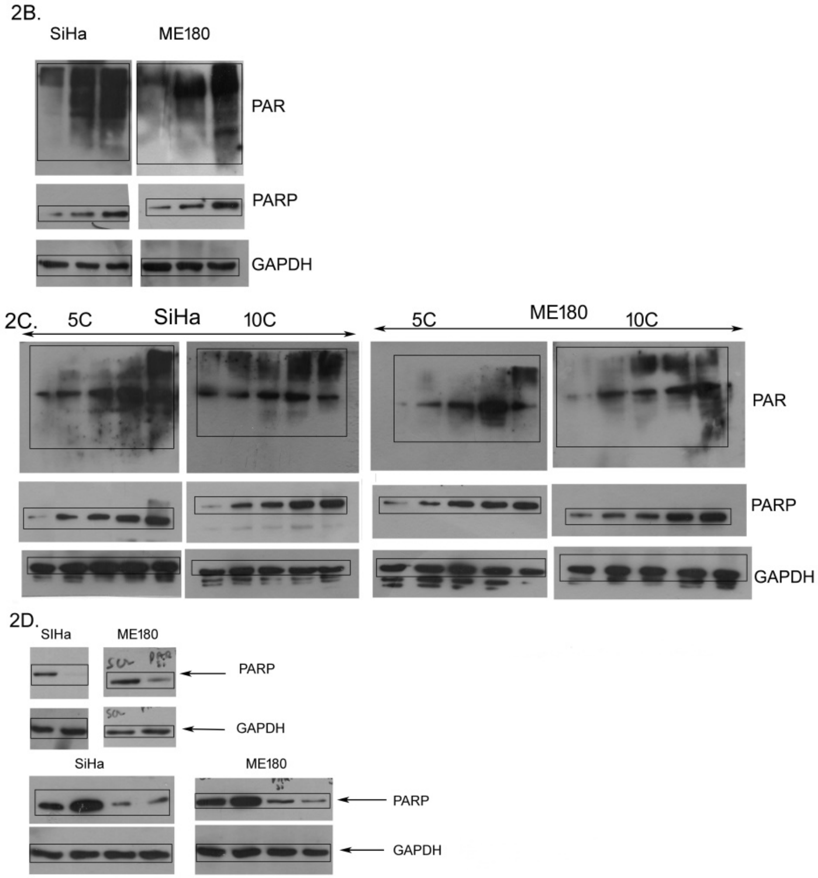

Supplement: Supplementary file 1 — Supplementary Information [file 41598_2017_13232_MOESM1_ESM.pdf]
